# Galectin Binding to Neo-Glycoproteins: LacDiNAc Conjugated BSA as Ligand for Human Galectin-3

**DOI:** 10.3390/biom5031671

**Published:** 2015-07-24

**Authors:** Sophia Böcker, Dominic Laaf, Lothar Elling

**Affiliations:** Laboratory for Biomaterials, Institute for Biotechnology and Helmholtz-Institute for Biomedical Engineering, RWTH Aachen University, Pauwelsstr. 20, 52074 Aachen, Germany; E-Mails: s.boecker@biotec.rwth-aachen.de (S.B.); d.laaf@biotec.rwth-aachen.de (D.L.)

**Keywords:** neo-glycoproteins, galectin-3, multivalency, LacDiNAc, chemo-enzymatic synthesis

## Abstract

Carbohydrate-lectin interactions are relatively weak. As they play an important role in biological recognition processes, multivalent glycan ligands are designed to enhance binding affinity and inhibitory potency. We here report on novel neo-glycoproteins based on bovine serum albumin as scaffold for multivalent presentation of ligands for galectins. We prepared two kinds of tetrasaccharides (*N*-acetyllactosamine and *N*,*N*-diacetyllactosamine terminated) by multi-step chemo-enzymatic synthesis utilizing recombinant glycosyltransferases. Subsequent conjugation of these glycans to lysine groups of bovine serum albumin via squaric acid diethyl ester yielded a set of 22 different neo-glycoproteins with tuned ligand density. The neo-glycoproteins were analyzed by biochemical and chromatographic methods proving various modification degrees. The neo-glycoproteins were used for binding and inhibition studies with human galectin-3 showing high affinity. Binding strength and inhibition potency are closely related to modification density and show binding enhancement by multivalent ligand presentation. At galectin-3 concentrations comparable to serum levels of cancer patients, we detect the highest avidities. Selectivity of *N*,*N*-diacetyllactosamine terminated structures towards galectin-3 in comparison to galectin-1 is demonstrated. Moreover, we also see strong inhibitory potency of our scaffolds towards galectin-3 binding. These novel neo-glycoproteins may therefore serve as selective and strong galectin-3 ligands in cancer related biomedical research.

## 1. Introduction

Carbohydrates play an important role in many biological processes as they act as recognition motifs for lectins and receptors triggering cellular signals and signal cascades [1,2,3,4,5,6,7,8]. Interactions of lectins with carbohydrate ligands are of a rather weak nature. For glycoproteins and glycolipids, binding strength may be drastically increased by presentation of multiple glycan ligands in so-called glyco clusters [9,10,11,12]. Therefore, multivalent glycan ligands are designed in drug research and anti-cancer therapy to enhance binding affinity and inhibitory potency. Multivalent carbohydrate presentation is mostly achieved by chemical modification of different scaffolds that define arrangement and orientation of the saccharides [13,14,15,16,17,18,19,20,21,22,23,24]. The reason for binding enhancement is discussed as chelate cooperativity [25,26], which describes interplay of binding sites for enhanced binding to their closely neighbored ligands presented in a multivalent system. These effects are found in many carbohydrate and non-carbohydrate related systems [27,28,29,30,31].

Here, we use serum albumin as scaffold for multivalent glycan presentation for lectin binding. Albumin is a highly abundant non-glycosylated serum protein and the protein of choice because it contains multiple lysine residues which are easily addressable for chemical conjugation methods [32,33,34]. Neo-glycoproteins are in general synthesized by chemical conjugation of glycans to native proteins [35,36]. They can be specifically designed for lectin binding studies by attachment of various types of glycan epitopes and by their glycosylation density [37,38,39].

Galectins, β-galactoside binding lectins, mediate most of their functions by galectin–glycan interactions [4,40,41]. They play an important role in a variety of biological processes including cancer progression and immune response [42,43,44,45,46,47,48]. Here, galectins can trigger apoptosis, metastasis and angiogenesis. Galectin-3 (Gal-3) is upregulated in many tumor cells extra- and intracellularly [49,50] and therefore a target for tumor diagnostic and therapy. It is the only member of the chimera-type galectin family with a conserved C-terminal carbohydrate recognition domain (CRD) and an N-terminal non-lectin domain [41]. Numerous studies have been performed to define glycan epitopes and find low molecular weight inhibitors for Gal-3 [51,52,53]. It is assumed that Gal-3 assembles as pentamers (via the N-terminal domain) or oligomers (via the C-terminal domain) upon binding to its carbohydrate ligand [54,55]. Thus, multivalent presentation of ligands plays a role in effective binding of Gal-3 [56,57,58,59].

The LacDiNAc (GalNAcβ1,4GlcNAc) epitope is overexpressed in certain parasites, but has a rather low abundancy in mammalian cells [60]. Gal-3 was found to bind to LacDiNAc and mediate immune recognition [60]. In human cancer progression, cancer type dependent up- or downregulation of LacDiNAc expression was observed [61,62]. Additionally, it was shown that the LacDiNAc epitope is present on the gastric mucin and recognized by an adhesin from *Helicobacter pylori* [63,64] which may play a role for bacterial colonization in the gastric mucosa.

Recently, we could confirm LacDiNAc as a selective ligand for Gal-3 compared to galectin-1 (Gal‑1) [65]. The inhibitory effect of divalent LacDiNAc for Gal-3 binding to asialofetuin glycoprotein, a model ligand for galectins, was weakly dependent on the linker length. However, a multivalent effect was not observed. In our previous studies, we found that the poly-LacNAc ([3Galβ1,4GlcNAcβ1]_n = 2 – 4_) oligomers are preferentially bound by Gal-3 [66]. We concluded that multivalent presentation of a tetrasaccharide glycan structure with a LacDiNAc epitope on a suitable scaffold should lead to increased inhibition of Gal-3 binding to glycoproteins.

We here report on the synthesis of two types of bovine serum albumin (BSA) based neo-glycoproteins carrying the tetrasaccharide structures LacNAc-LacNAc and LacDiNAc-LacNAc, respectively, with various degrees of multivalency. First, the glycans carrying an amino terminated linker at their reducing end were synthesized by multi-step chemo-enzymatic synthesis as previously reported [65,67]. Chemical conjugation to lysine residues of BSA was accomplished by homobifunctional amino-reactive linker squaric acid diethyl ester enabling crosslinking via primary amino groups [68,69,70,71,72,73,74,75]. Variation of the molar ratios of glycan with respect to lysine residues resulted in the synthesis of 11 neo-glycoproteins of both types with different degrees of glycan modification. The multivalent neo-glycoproteins were finally evaluated in binding studies with human Gal-3 and Gal-1 to determine their binding and affinity properties as well as inhibitory potential.

## 2. Results and Discussion

Here, we present neo-glycoproteins with varying glycosylation density based on BSA and their application in galectin binding studies. For our purposes, the oligomers LacNAc-LacNAc and LacDiNAc-LacNAc were synthesized *de novo* chemo-enzymatically. Decoration of BSA was accomplished by a two-step conjugation reaction using squaric acid diethyl ester as a linker. Irrespective of the accessibility, BSA can be decorated with up to 60 glycans per molecule due to the presence of 60 lysine residues. The synthesized neo-glycoproteins are tested as ligands for human Gal‑3 and Gal-1.

### 2.1. Chemo-Enzymatic Synthesis of LacNAc-LacNAc and LacDiNAc-LacNAc

Glycosyltransferases and activated nucleotide sugars as donor substrate were applied in a consecutive synthesis for attachment of monosaccharide residues to GlcNAc-linker-*t*Boc (**1**) (Scheme I). By sequential elongation of **1** using recombinantly expressed and purified β4GalT [76] and β3GlcNAcT [67,77], compounds **2** and **3** (both 100% conversion) were obtained. Compound **3** was further elongated using either β4GalT or β4GalTY284L [65,78] for the synthesis of Gal- or GalNAc-terminated oligomers **4** and **5**, respectively. In contrast to our previously published method [65], a three-fold molar excess of UDP-GalNAc and appropriate amount of β4GalTY284L (75 mU/mL) turned out to be crucial to obtaining a quantitative yield for **5**. After purification yields of **4** (90.6%, 45.30 µmol LacNAc-LacNAc) and **5** (98.3%, 138.6 µmol LacDiNAc-LacNAc) were determined via HPLC analysis. Integrity and purity of 4 and 5 were confirmed via LC-MS (Figure S1**a,b**). Deprotection of **4** and **5** carrying *tert*-butyloxycarbonyl (*t*Boc) protecting group was carried out under acidic conditions yielding **6** (85.0%, 42.5 µmol) and 7 (96.1%, 133.19 µmol).

In conclusion, multi-step chemo-enzymatic synthesis and deprotection provided two pure tetrasaccharides (LacNAc-LacNAc and LacDiNAc-LacNAc) in good yields.

**Scheme I:**
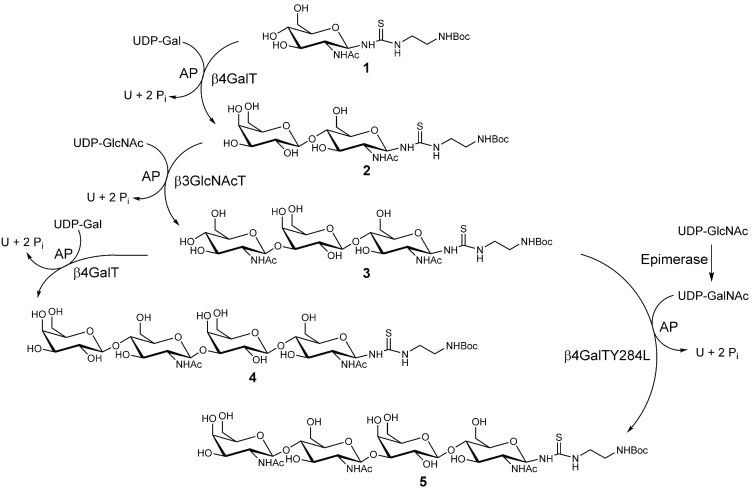
Chemo-enzymatic synthesis of tetrasaccharides **4** and **5** using recombinant glycosyltransferases for complete conversions.

### 2.2. Synthesis and Purification of Squaric Acid Monoamide Esters

Consecutive attachment of two different ligands with the same functional group is the most attractive feature of squaric acid diethyl ester (**8**) [72]. Thus, **8** offered ideal chemical properties for BSA decoration with LacNAc-LacNAc or LacDiNAc-LacNAc. Amino group providing oligomers **6** and **7** were conjugated to **8** forming squaric acid monoamide esters **9** and **10** (Scheme II). Formation of squaric acid bisamide was suppressed by constant pH 7 and the presence of triethylamine (Et_3_N) as external base. Complete conversion of **6** and **7** could only be achieved by applying a four-fold molar excess of **8** and equal amounts of Et_3_N. The purity of the products **9** and **10** had highest priority as trace impurities of **8** would lead to protein cross-linking reactions during the second conjugation step. Thus, removal of residual **8** was achieved using preparative HPLC. Additionally, LC‑MS results (Figure S1**c,d**) confirmed integrity and purity of compounds **9** and **10**. Molecular masses of 972.3 *m*/*z* and 1013.6 *m*/*z*, respectively, were found for **9** and for **10**.

In summary, overall yields of pure **9** (77.8%) and **10** (86.3%) were determined after chemo-enzymatic synthesis, deprotection and conjugation to **8**.

### 2.3. Synthesis and Analysis of Neo-Glycoproteins

Aqueous solution with increased pH was utilized as reaction buffer for squaric acid bisamide production, as recommended [70,72,79]. In order to generate neo-glycoproteins presenting variable numbers of sugar moieties, different concentrations of **9** or **10** were applied for the coupling process (Scheme II). Active charcoal delipidated BSA was dissolved in borate buffer (pH 9.0) and ratios of **9** or **10** ranging between 0.025 and 1.5 with respect to BSA lysine residues were adjusted. Thus, neo-glycoproteins **11a–k** and **12a–k** with variable numbers of LacNAc-LacNAc and LacDiNAc-LacNAc, respectively, were obtained after an incubation period of six days.

**Scheme II:**
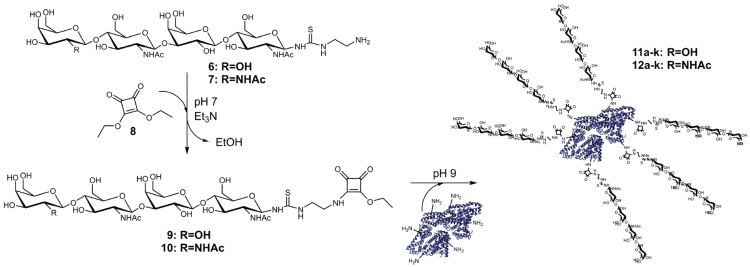
Two-step neo-glycoprotein synthesis.

Trinitrobenzene sulfonic acid (TNBSA) was utilized for quantification of free lysine residues of BSA. Nucleophilic character of TNBSA as nitrobenzene derivative is well known for precise labeling of primary amines. The photometric measurement of the resulting *N*‑trinitrophenylamine at 420 nm was demonstrated as the suitable wavelength for colorimetric quantification of free lysine residues [80]. In order to minimize consumption of valuable neo-glycoproteins experimental setup of the TNBSA assay was optimized. Lysine hydrochloride and BSA were utilized for highly accurate calibration (Figure S2) of the TNBSA assay. Finally, the number of unmodified (free) lysine residues of the neo-glycoproteins **11a–k** and **12a–k** was determined by the optimized TNBSA assay as shown in Table 1. Molecular weights of the glycoproteins were then calculated according to the number of attached LacNAc-LacNAc or LacDiNAc-LacNAc glycans (amidated lysine residues). Increased ratios of **9** or **10** with respect to lysine residues of BSA led to an increasing modification density as more lysine residues were amidated.

For **11a–d** and **12a–d** the coupling efficiencies of squaric acid monoamide esters **9** and **10** were between 73.0% and 94.4%. However, at higher molar ratios coupling efficiency was only 32.2% for **9** and 30.6% for **10** yielding BSA based neo-glycoproteins **11i** and **12i**, respectively. Possible reasons for this observed limitation may be low accessibility of some of the lysine residues and hydrolysis of the squaric acid monoamide esters [72,79]. In summary, different amounts of **9** or **10** were attached to BSA reaching numbers for modified lysine residues between 2 and 29 per BSA molecule. Our results are in accordance with published data, where lactose conjugated BSA with maximal 30 glycation sites was obtained at lactose-BSA ratios of 20:1 [75]. In contrast, we reach this grade of modification already with a 1.5-fold molar excess of squaric acid monoamide esters (**9** or **10**) despite their approximately doubled molecular masses with respect to lactose.

**Table 1:** Glycosylation densities and resulting molecular weights of BSA based neo-glycoproteins **11a–k** and **12a–k**. The molecular weights (MW) of BSA based neo-glycoproteins were calculated according to the number of attached LacNAc-LacNAc (**11a–k**) and LacDiNAc-LacNAc (**12a–k**) glycans using TNBSA assay.

Compound	Unmodified lysine residues [mol/mol BSA]	Amidated lysine residues [mol/mol BSA]	MW [kDa]	Compound	Unmodified lysine residues [mol/mol BSA]	Amidated lysine residues [mol/mol BSA]	MW [kDa]
BSA	60.0 ± 0.027	0.0	66.4	BSA	60.0 ± 0.027	0.0	66.4
**11a**	58.4 ± 0.016	1.6	67.9	**12a**	58.3 ± 0.012	1.7	68.1
**11b**	56.2 ± 0.005	3.8	70.0	**12b**	57.8 ± 0.005	2.2	68.6
**11c**	53.9 ± 0.013	6.1	72.2	**12c**	54.1 ± 0.012	5.9	72.2
**11d**	50.0 ± 0.040	10.0	75.9	**12d**	51.2 ± 0.012	8.8	75.1
**11e**	45.6 ± 0.015	14.4	80.0	**12e**	45.9 ± 0.046	14.1	80.3
**11f**	40.6 ± 0.027	19.4	84.7	**12f**	38.7 ± 0.091	21.3	87.4
**11g**	35.8 ± 0.079	24.2	89.3	**12g**	35.6 ± 0.115	24.4	90.4
**11h**	34.0 ± 0.009	26.0	90.9	**12h**	35.0 ± 0.066	25.0	91.0
**11i**	31.0 ± 0.130	29.0	93.8	**12i**	32.5 ± 0.097	27.5	93.5
**11j**	52.5 ± 0.012	7.5	73.5	**12j**	52.6 ± 0.018	7.4	73.7
**11k**	42.2 ± 0.084	17.8	83.2	**12k**	42.0 ± 0.060	18.0	84.1

**Figure 1:**
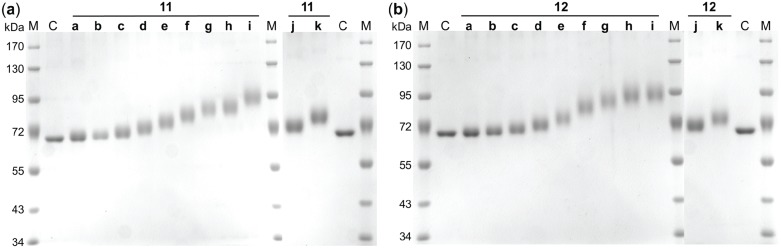
SDS-PAGE analysis of neo-glycoproteins. Electrophoretic mobility of BSA based neo-glycoproteins presenting (**a**) LacNAc-LacNAc (**11a–k**) and (**b**) LacDiNAc-LacNAc (**12a–k**) is compared to unmodified BSA (lane C). M—Protein size standard, C—unmodified BSA, **11a–k**—LacNAc-LacNAc conjugated BSA (1 µg/lane), **12a–k**—LacDiNAc-LacNAc conjugated BSA (1 µg/lane).

Ultrafiltration was used for removal of residual **9** or **10** to exclude effects on galectin binding assays. Pure neo-glycoproteins **11a–k** (yields of 45.6%–68.2%) and **12a–k** (yields of 61.7%–87.7%) were finally obtained (Figure 1). To gain higher modification densities, a fed-batch instead of batch mode for chemical conjugation might be an alternative to overcome squaric acid monoamide ester hydrolysis. Neo-glycoproteins **11j–k** and **12j–k** featuring almost identical modification degrees were synthesized for subsequent comparison of LacNAc-LacNAc conjugated BSA (**11j–k**) and LacDiNAc-LacNAc conjugated BSA (**12j–k**) in galectin inhibition assay.

TNBSA assay results (Table 1) indicated consistent increase of theoretical molecular weights of neo-glycoproteins **11a–k** and **12a–k**. For further evaluation, apparent molecular weights of neo-glycoproteins were determined by reducing polyacrylamide gel electrophoresis (SDS-PAGE) shown in Figure 1. As molecular weight of BSA (66.4 kDa) could be confirmed via SDS-PAGE, gentle active charcoal treatment for removal of lipids does obviously not influence protein integrity. Moreover, Figure 1 provides direct evidence for molecular weight shift of neo-glycoproteins towards higher values. As expected, the mass differences of **11a–k** or **12a–k** compared to unmodified control (lane C) increase with higher initial concentrations of substances **9** or **10**. Neo-glycoproteins **11a–k** and **12a–k** do not appear as distinct bands while smearing effect increases with size. Smearing appearance of glycosylated BSA can be due to the statistical character of the coupling reaction and thus presence of BSA molecules with different degrees of modification. Moreover, hydrophilic sugar chains are known to lack interaction with detergents like SDS [81]. Consequently, detergent induced development of linearized proteins is prevented proportionally to the amount of glycan moieties. Nevertheless, molecular weights of neo-glycoproteins are evaluated. For this purpose, retardation factors (R_f_) are determined for each sample (**11a–k** and **12a–k**). Molecular weight differences with regard to unmodified BSA were calculated and are summarized in Table 2.

**Table 2:** Molecular weight differences (∆MW) of neo-glycoproteins **11a–k** and **12a–k** compared to unmodified BSA. Values for retardation factor (R_f_) were calculated based on electrophoretic mobility of samples resulting from SDS-PAGE analysis (Figure 1). Measured R_f_ values were transformed to molecular weights by linear regression (y = mx + b; y—log MW; m—slope; x—R_f_; b—y-intercept). Differences between the calculated molecular weights of neo-glycoproteins and those of unmodified BSA are compared with data determined by TNBSA assay.

Compound	TNBSA ΔMW [kDa]	SDS-PAGE ΔMW [kDa]	Compound	TNBSA ΔMW [kDa]	SDS-PAGE ΔMW [kDa]
BSA	0.0	0.0	BSA	0.0	0.0
**11a**	1.5	1.3	**12a**	1.7	1.5
**11b**	3.6	3.0	**12b**	2.2	2.5
**11c**	5.8	5.0	**12c**	5.8	4.6
**11d**	9.4	8.2	**12d**	8.7	7.3
**11e**	13.6	12.1	**12e**	13.9	11.7
**11f**	18.3	17.4	**12f**	21.0	19.0
**11g**	22.8	22.4	**12g**	24.0	22.2
**11h**	24.5	24.3	**12h**	24.6	25.5
**11i**	27.3	27.6	**12i**	27.1	28.2
**11j**	7.1	7.0	**12j**	7.3	7.1
**11k**	16.8	15.0	**12k**	17.7	15.6

Coupling reactions of squaric acid monoamide esters **9** or **10** lead to mass increase of 0.943 kDa or of 0.984 kDa per amidated lysine residue. Results from biochemical TNBSA assay and SDS-PAGE provide direct evidence for molecular weight shift of neo-glycoproteins **11a–k** and **12a–k**. Both approaches are suitable analysis methods as approximately equal differences of molecular weight are identified. Most molecular weights indicated by SDS-PAGE are slightly smaller compared to those resulted from TNBSA assay. The analysis of diffuse bands leads to certain inaccuracy of R_f_ values.

In conclusion, BSA based neo-glycoproteins **11a–k** and **12a–k** were analyzed using different experimental setups. Maximal 29 of 60 lysine residues were demonstrated to be accessible by squaric acid monoamide esters by a tuned molar excess (1.5-fold) of those. Neo-glycoproteins appeared as smeary bands on reducing gel due to glycan proportions. However, shifts of molecular weights already indicated by TNBSA assay could be confirmed by SDS-PAGE. Clean BSA based neo-glycoproteins varying in the degree of presented oligosaccharide moieties were synthesized. Consequently, samples **11a–k** and **12a–k** were used for ELISA-type galectin binding tests.

### 2.4. Comparison of Galectin-3 and Galectin-1 Binding to Neo-Glycoproteins

**Figure 2:**
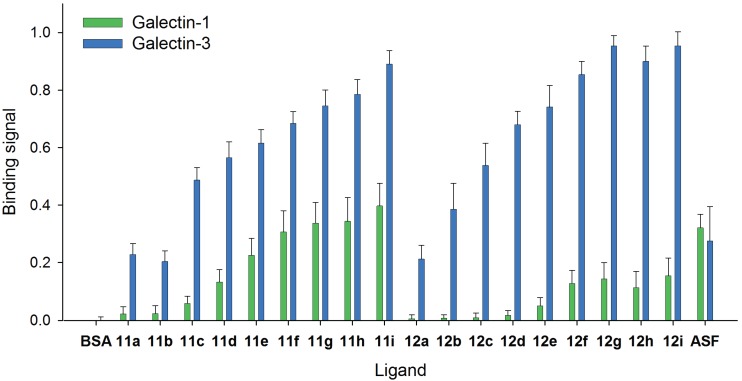
Comparison of galectin-1 and galectin-3 binding to immobilized neo-glycoproteins **11a–i** and **12a–i**. For neo-glycoproteins, binding signals of 1 µM galectin-1 (■) and 1 µM galectin-3 (■) are compared. Galectin binding to immobilized neo-glycoproteins as well as to unmodified BSA is shown. All galectin-3 binding signals are significantly higher than those of galectin-1 (*p* < 0.002).

The selective binding of human Gal-1 and Gal-3 to the synthesized neo-glycoproteins was investigated by ELISA-type assay using galectin concentration of 1 µM (Figure 2). Both galectins show no binding to unmodified BSA. Whereas binding signals of both galectins to the standard glycoprotein asialofetuin (ASF) are similar, Gal-3 shows higher binding to neo-glycoproteins compared to Gal-1. At low modification degrees of LacNAc-LacNAc conjugated BSA (**11a–d**), Gal-3 binds up to 10 times higher, whereas at higher glycan numbers (**11e–i**), Gal-3 reaches two-fold higher binding signals than Gal-1 (Figure 2 and Table S1). The binding differences between Gal-1 and Gal-3 are proven to be significant with *p* < 0.002. Our data confirm previous studies that galactose terminated tetrasaccharides and oligosaccharides have higher selectivity for binding of Gal-3 compared to Gal-1 [52,66,82,83].

Most importantly, the difference between Gal-3 and Gal-1 binding is more distinct regarding LacDiNAc-LacNAc conjugated BSA (Figure 2 and Table S1). The binding of Gal-3 to neo-glycoproteins **12a–d** is up to 60-fold higher, and to BSA with higher glycan densities (**12e–i**) seven-fold higher when compared to Gal-1. The smaller difference between the binding potencies of both galectins to highly modified neo-glycoproteins is probably caused by reaching the maximal binding density of Gal-3 to neo-glycoproteins as well as increased binding of Gal-1 if multiple ligands are presented. Gal-1 is known to recognize terminal and not internal galactose [84,85], but in the present and earlier studies, weak binding to internal galactose occurred [66,86,87]. Due to the fact that Gal-1 does not bind to LacDiNAc [60,65,83,84], weak binding of Gal-1 to LacDiNAc-LacNAc conjugated BSA is based on recognizing the internal LacNAc unit since multiple ligands are presented.

In conclusion, neo-glycoproteins modified either with LacNAc-LacNAc or LacDiNAc-LacNAc show higher selectivity for Gal-3 compared to Gal-1. LacDiNAc-LacNAc conjugated BSA exhibits highly distinct selectivity for Gal-3, especially at low modification degrees (**12a–d**). For putative application, e.g., anti-cancer therapy or imaging, Gal-3 could solely be addressed using low modified LacDiNAc-LacNAc conjugated BSA.

### 2.5. Galectin-3 Binding to Neo-Glycoproteins at Different Galectin Concentrations

LacNAc and LacDiNAc epitopes are recognized by human Gal-3 with preferential binding to LacDiNAc [65]. Moreover, we identified in previous studies the LacNAc-LacNAc tetrasaccharide as the preferable Gal-3 ligand [66] pointing out that the glycans **4** and **5** are suitable candidates for developing multivalent neo-glycoproteins. BSA with varying numbers of **4** and **5** were analyzed for their binding characteristics as ligands for human Gal-3.

**Figure 3:**
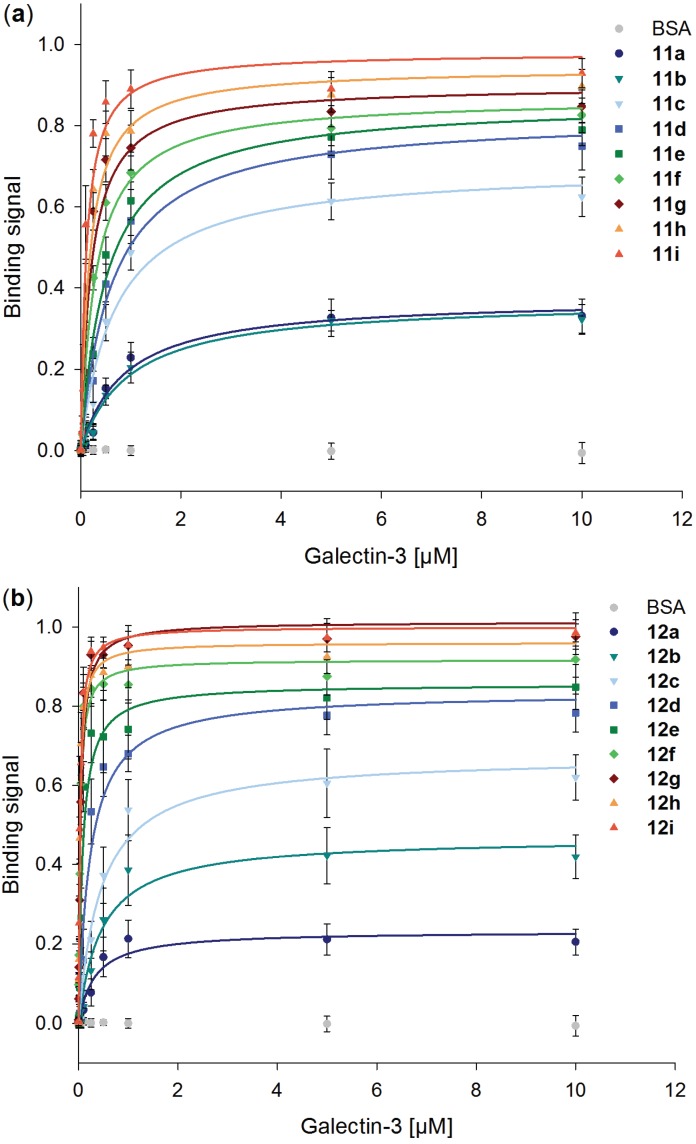
Binding of human galectin-3 to different neo-glycoproteins. Different amounts of galectin-3 were incubated on immobilized neo-glycoproteins (5 pmol per well) presenting (**a**) LacNAc-LacNAc (**11a–i**) and (**b**) LacDiNAc-LacNAc (**12a–i**) in an ELISA-type assay. Binging signals are plotted against galectin-3 concentration. The curves were fitted using Sigma Plot software.

In Figure 3, binding signals of Gal-3 on immobilized neo-glycoproteins increase with higher modification densities. The results confirm Gal-3 binding to both types of BSA based neo-glycoproteins and no binding to unmodified BSA. However, significant increases of saturated binding signals are detected for BSA based neo-glycoproteins up to a modification degree of approximately 20 glycans per BSA, neo-glycoproteins **11a–g** and **12a–f**, respectively. Binding of Gal-3 hardly improves with increasing numbers of glycans above these modification degrees which may be due to approaching the maximal Gal-3 steric occupancy per molecule. The maximal binding signal of Gal-3 is for **11a–c** and **12a–c** in the same range but is slightly higher for **12d–i** compared to **11d–i** which may hint at better binding of Gal-3 to the LacDiNAc epitope.

**Figure 4:**
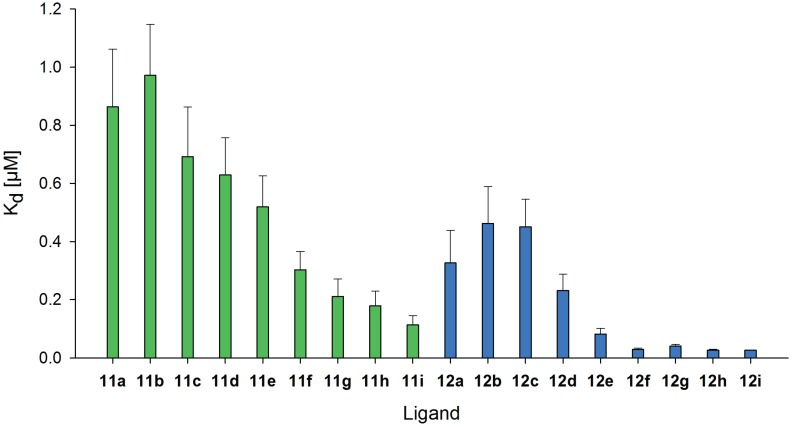
K_d_ values of human galectin-3 binding to different neo-glycoproteins. The K_d_ values enable the comparison of the affinity of galectin-3 to the tested neo-glycoproteins modified with LacNAc-LacNAc **11a–i** (■) and LacDiNAc-LacNAc **12a–i** (■). K_d_ values in [µM] were calculated using Sigma Plot software.

The binding curves were further analyzed and used for calculation of the K_d_ values for Gal-3 binding (Figure 4 and Table S2). In general, LacDiNAc-LacNAc conjugated BSA shows better binding of Gal-3 compared to LacNAc-LacNAc conjugated BSA resulting in general lower K_d_ values for all modification degrees **12a–i**. Significant differences between both types of neo-glycoproteins are obvious reflecting different selectivity of Gal-3 for the glycans. For binding of Gal-3 to **11a–i** K_d_ values decrease more gradually reaching the lowest K_d_ value of 0.1 µM for **11i** (Table S2). A significant drop of the K_d_ value is observed for the neo-glycoproteins **11f** and **11i** with about 19 and 29 LacNAc-LacNAc glycans, respectively. In comparison, K_d_ values are already significantly lowered for **12d–f** with about nine, 14 and 21 LacDiNAc-LacNAc glycans, respectively, reaching a K_d_ value for Gal-3 in the nanomolar range (3 × 10^−8^ M for **12f**). These data emphasize high selectivity of Gal-3 for LacDiNAc-LacNAc glycans.

**Figure 5:**
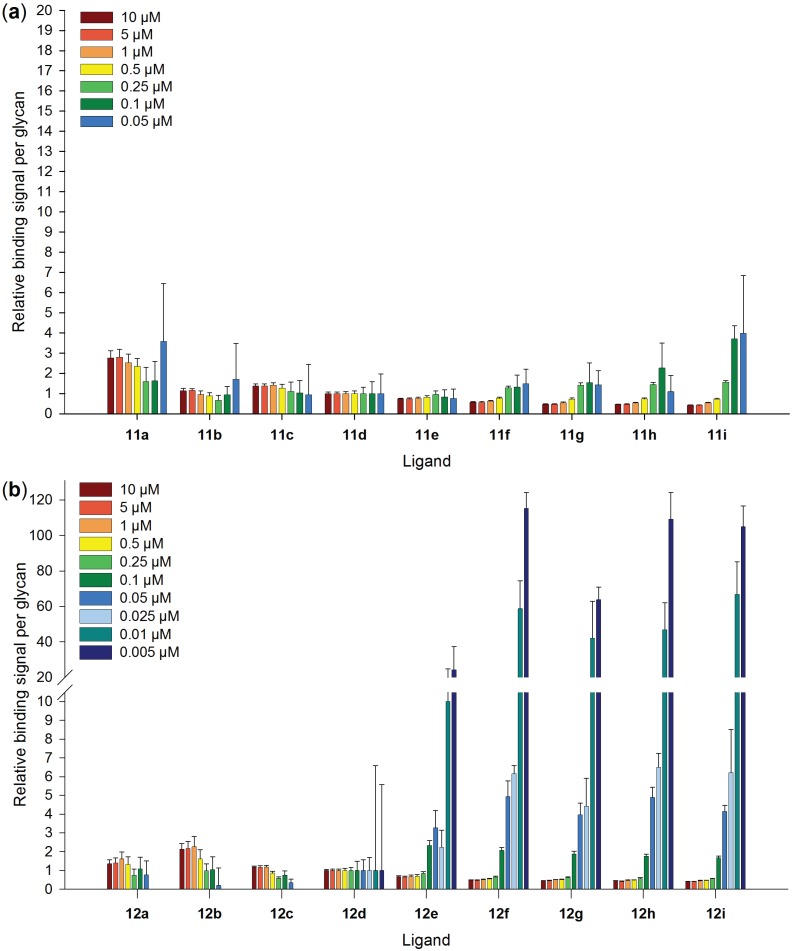
Binding signals of human galectin-3 in relation to one binding site for BSA based neo-glycoproteins. Relative signals of galectin-3 binding to neo-glycoproteins with (**a**) LacNAc-LacNAc and (**b**) LacDiNAc-LacNAc at galectin-3 concentration ranging from 0.005–10 µM are shown. Values of binding enhancement result from binding signals per glycan in relation to the galectin-3 binding per glycan for **11d** and **12d**, respectively.

In order to evaluate multivalent effects, the binding signal per glycan ligand was calculated for all applied Gal-3 concentrations and was related to **11d** and **12d**, respectively (Figure 5 and Table S3). For **11a–h** and **12a–c**, binding signals are only detectable for Gal-3 concentrations from 0.05 µM (Figure S3). In contrast, for LacDiNAc-LacNAc conjugated BSA modification densities of at least nine glycans per BSA (**12d**) are sufficient to reach binding signals of Gal-3 at concentrations even below 0.05 µM. Thus, binding signals per glycan for **11d** and **12d** are used as the benchmark for calculating binding potencies.

Enhancement of binding by multivalent presentation is not observed for LacNAc-LacNAc conjugated BSA. Binding signals of Gal-3 to **11a–i** in relation to one binding site reach nearly the same level, in the used concentration range. In contrast, Gal-3 shows enhanced avidity for binding to neo-glycoproteins **12e–i** at concentrations below 0.05 µM with highest multivalent effects for 0.005 µM. Up to 100 times higher binding in relation to one binding site is observed for modification densities above 20 glycans per BSA. However, at Gal-3 concentrations above 0.1 µM, the binding signals in relation to one glycan are for higher modified BSA even lower than for weak glycosylated BSA. This is possibly due to hindered accessibility of all binding sites in the presence of Gal-3 excess. Consequently, multivalent ligand presentation on BSA can induce multivalent effects depending on the ligand, the valency and the galectin concentration. Here, only LacDiNAc-LacNAc conjugated BSA with modification degrees above eight glycans per BSA enhances the avidity of Gal-3 at concentrations below 0.05 µM. Multivalent effects were also found for Gal-3 using *N-*glycan modified human serum albumin synthesized by click-chemistry [88]. The multivalent neo-glycoprotein carrying eight biantennary *N*-glycans showed a 30-fold increase in affinity per glycan unit in comparison to the monovalent reference. For Gal-1 it was reported that BSA based neo-glycoproteins with up to eight conjugated glycans per BSA did not show enhanced avidity [84]. The monovalent recognition of the glycans by Gal-1 was explained by the high molecular weight of BSA and therefore the long distance between the glycans.

Mostly, Gal-3 binding was investigated for multivalent ligands based on chemical scaffolds like calixarenes, dendrimers or quantum dots [58,59,89,90,91]. Comparison of our results with findings for these synthetic scaffolds is difficult but can be drawn with regard to the glycan type and number. With multivalent LacNAc modified quantum-dots presenting 108 glycans, a 92-fold higher affinity towards Gal-3 in comparison to soluble LacNAc was shown using surface plasmon resonance spectroscopy [58]. With respect to one ligand, we gain higher enhancement of Gal-3 binding to BSA based neo-glycoproteins modified with a much smaller number of glycans, namely at least 14 LacDiNAc-LacNAc glycans (**12f**).

Neo-glycoproteins enhancing the binding potency of low concentrated Gal-3 by a factor of 100 may be promising inhibitors for anti-cancer therapy. The expression of Gal-3 is upregulated in different tumor cells and induces tumor progression and metastasis [42,43,44,45,46,47,48,92]. The serum level of Gal-3 in patients with cancer is likewise significantly increased compared with healthy individuals [93,94,95]. Therefore, interfering with Gal-3 by applying effective inhibitors can reduce the angiogenic and metastatic potential of tumor cells [96,97,98,99]. Multivalent systems, e.g., based on dendrimer scaffolds, are of high interest as they induce cluster formation and exhibit enhanced inhibitory potencies [53,91,100]. In the present study, we have generated neo-glycoproteins which are effective for multivalent binding at high modification degrees (**12f–i**) with the Gal-3 selective epitope LacDiNAc-LacNAc. The highest multivalent effects occur at Gal-3 concentrations of 5–10 nM (130–260 ng/mL) which is in the range of Gal-3 level in human serum of cancer patients (mean value = 320 ng/mL). This is about five times higher than in healthy individuals (mean value = 62 ng/mL) [95]. Hence, in roughly that concentration range, Gal-3 inhibition should reduce tumor aggressiveness making these LacDiNAc-LacNAc conjugated BSA promising candidates for anti-cancer therapy. *In vivo* applications are possible, because BSA based neo-glycoproteins were already applied to mice, rats and rabbits [101,102].

In conclusion, BSA-based neo-glycoproteins are high-affinity ligands for Gal-3 with higher binding for increasing modification densities. At Gal-3 concentrations below 0.05 µM, up to 100-fold enhanced avidity is shown for neo-glycoproteins presenting more than 14 LacDiNAc-LacNAc glycans.

### 2.6. Inhibitory Potency of Neo-Glycoproteins

**Figure 6:**
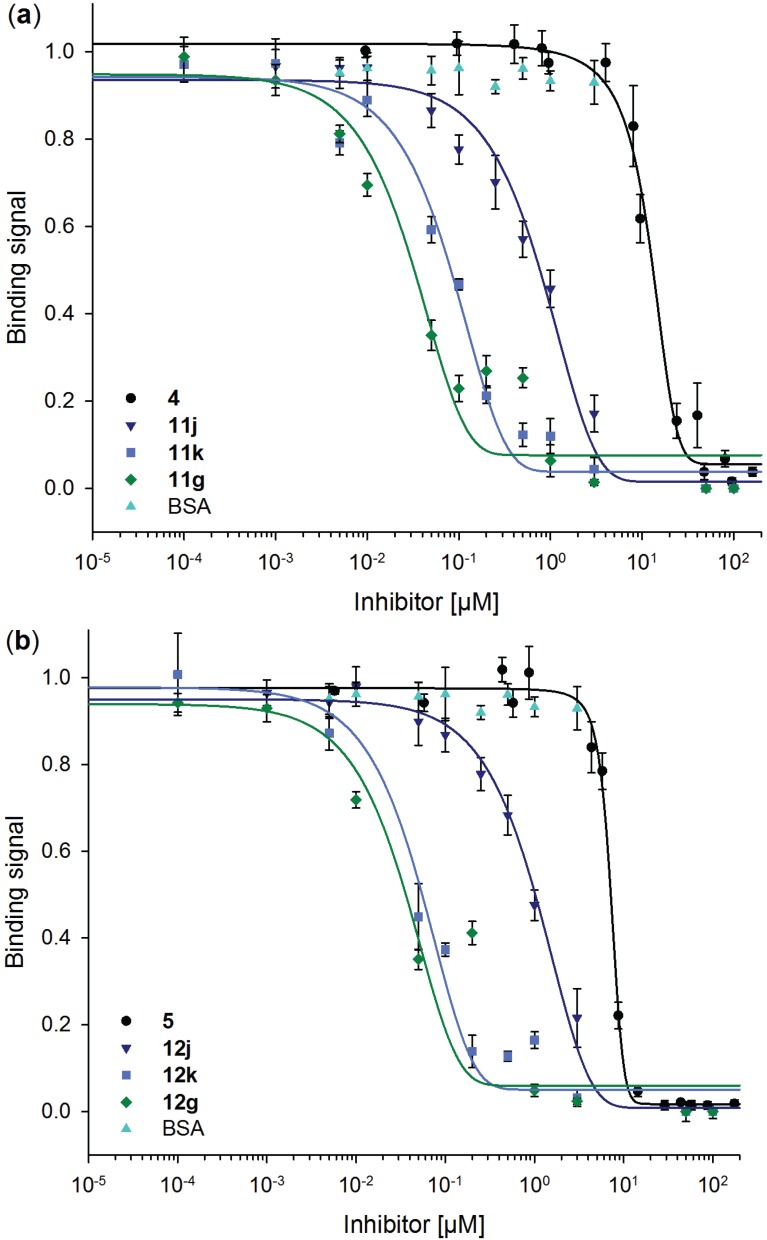
Competitive inhibition of galectin-3 binding to ASF with free tetrasaccharides and neo-glycoproteins. Residual binding of 5 µM galectin-3 to ASF in the presence of variable concentrations of (**a**) LacNAc-LacNAc (**4**) and LacNAc-LacNAc conjugated BSA (**11j**, **11k** and **11g**) and (**b**) LacDiNAc-LacNAc (**5**) and LacDiNAc-LacNAc conjugated BSA (**12j**, **12k** and **12g**) is shown. Unmodified BSA has no influence on the galectin-3 binding. Inhibition curves were fitted using Sigma Plot software.

**Figure 7:**
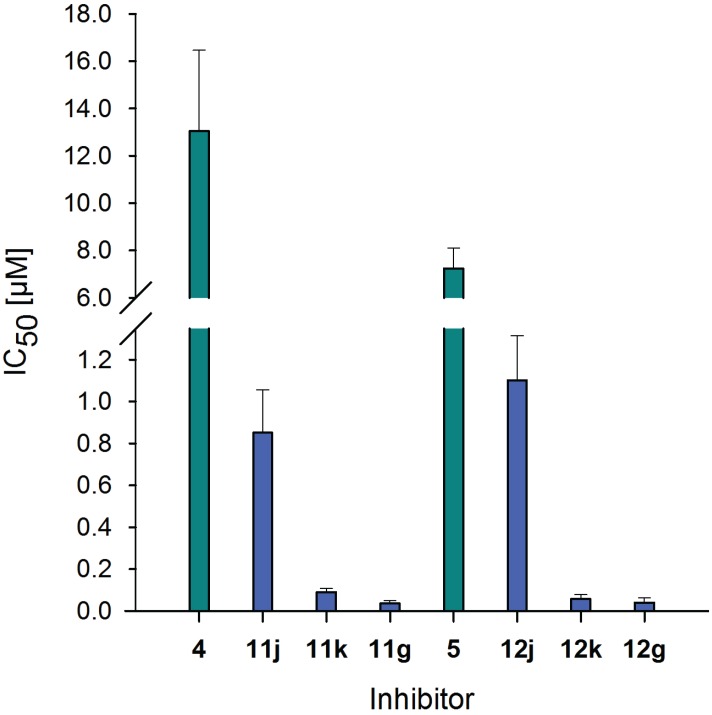
IC_50_ values of soluble tetrasaccharides and neo-glycoproteins. IC_50_ values of neo-glycoproteins **11j**, **11k**, **11g**, **12j**, **12k** and **12g** (■) are compared with those of free tetrasaccharides **4** and **5** (■) for 5 µM galectin-3 binding on ASF. Lower values indicate higher inhibitory potencies. IC_50_ values in [µM] were calculated using Sigma Plot software.

For possible biomedical application of these neo-glycoproteins, their ability to inhibit Gal-3 binding has to be proven. Thus, competitive inhibition assay was performed to investigate Gal-3 binding to immobilized ASF in the presence of neo-glycoproteins **11j**, **11k**, **11g** and **12j**, **12k**, **12g**, respectively. To compare inhibitory potencies with soluble oligosaccharides, **4** and **5** were also used in this assay. Plotting residual Gal-3 binding signals against inhibitor concentration provides the sigmoidal curves depicted in Figure 6 and are used for IC_50_ calculation (Figure 7 and Table 3).

Inhibition of Gal-3 binding by neo-glycoproteins is shown by a shift to lower inhibitor concentrations indicating higher inhibition potencies of ligands presented multivalently on a protein scaffold. IC_50_ values of **4** and **5** are significantly higher than those reached by the neo-glycoproteins (*p* < 0.002). With increasing glycosylation densities of the neo-glycoproteins, stronger inhibition of Gal-3 binding to ASF is observed. BSA modified with 18 and 24 binding sites (**11k**, **11g** and **12k**, **12g**) are obviously stronger inhibitors than BSA presenting 7.5 glycans (**11j** and **12j**) though only small differences exist between both higher modification degrees. Increased modification of BSA results in higher inhibition, but does not strengthen the inhibition proportionally. When comparing the tetrasaccharides LacDiNAc-LacNAc (**5**) and LacNAc-LAcNAc (**4**), **5** reveals a 1.5-fold higher inhibition of Gal-3 binding. This is based on better binding to LacDiNAc terminated glycan as already demonstrated [65].

**Table 3:** Inhibition and relative potencies of galectin-3 binding to ASF by soluble tetrasaccharides and neo-glycoproteins. Relative potencies are related to the corresponding tetrasaccharide and additionally to one binding site. Neo-glycoproteins show up to 15 times enhanced potencies per glycan compared to soluble tetrasaccharides.

Compound	IC_50_ [µM]	Relative potency	Relative potency per glycan
**4**	13.04 ± 3.43	1.0 ± 0.26	1.0 ± 0.26
**11j**	0.85 ± 0.21	15.3 ± 3.14	2.0 ± 0.42
**11k**	0.09 ± 0.02	145.2 ± 2.45	8.2 ± 0.14
**11g**	0.04 ± 0.01	367.8 ± 5.12	15.2 ± 0.21
**5**	7.23 ± 0.87	1.0 ± 0.12	1.0 ± 0.12
**12j**	1.10 ± 0.22	6.6 ± 1.42	0.9 ± 0.19
**12k**	0.06 ± 0.02	127.1 ± 2.56	7.1 ± 0.15
**12g**	0.04 ± 0.02	182.0 ± 4.06	7.5 ± 0.17

In this context, the evaluation of multivalent effects is relevant. Therefore, we chose neo-glycoproteins of low (**11j** and **12j**), medium (**11k** and **12k**) and high (**11g** and **12g**) modification degree used in this competitive inhibition assay and calculated relative potencies per glycan (Table 3). The improvement of the inhibitory potency by multivalent presentation is overall more pronounced for LacNAc-LacNAc conjugated BSA than for LacDiNAc-LacNAc conjugated BSA since the IC_50_ value for **4** is higher than for **5**. Thereby, the inhibitory potency can be more enhanced. The general presentation of glycans on BSA has little influence on the inhibitory potency indicated by only up to two times improved relative potency in relation to one binding site compared to **4** and **5**. Here, we can conclude that multivalent effects are not relevant for low glycosylation degrees of about 7.5 glycans per BSA (**11j**, **12j**). However, compounds **11j** and **12j** improve the inhibition of Gal-3 binding to ASF by a factor of 15 and 6.5, respectively. For medium and high modification densities (**11k**, **11g** and **12k**, **12g**), the inhibitory potency is drastically enhanced by factors of 130–370 related to the corresponding tetrasaccharide. In relation to one binding site, inhibitory potency is improved by **11k** four times and by **11g** 7.5 times in comparison to **11j**, and almost twice by **11g** compared to **11k**. Regarding LacDiNAc-LacNAc conjugation, inhibition potency is enhanced by **12k** by a factor of 7 per glycan compared to **12j**, whereas with **12g** scarcely any further improvement is gained. Higher inhibitory potency of LacDiNAc-LacNAc conjugated BSA over LacNAc-LacNAc conjugated BSA is not observed. In contrast, we see binding enhancement of Gal-3 to LacDiNAc-LacNAc presenting neo-glycoproteins with at least 14 glycans (**12e–i**) at Gal-3 concentrations below 0.05 µM (Figure 5b). The inhibition assay requires higher Gal-3 concentration (5 µM) due to weaker binding to ASF than to neo-glycoproteins. Therefore, equal inhibition potencies of both types of modifications are obtained.

However, we determined IC_50_ values below 100 nM for BSA based neo-glycoproteins modified with at least 18 glycans. Reaching such high inhibitory potencies is caused by binding enhancement due to protein chelation in the presence of multivalent ligands [11]. As Gal-3 forms oligomers involving multivalent ligands [54,55] it is prone to be a candidate for binding enhancement by multivalent effects. In contrast, Gal-3 binding to ASF was characterized by high affinity to the first LacNAc epitope of the triantennary *N*-glycan with increasing negative cooperativity for subsequent binding events [56]. However, lactose based dendrimers showed strong multivalent effects in a solid phase inhibition assay with ASF [103]. Gal-3 binding to monovalent lactose dendrimer was already about 10 times higher than to free lactose indicating interactions with the dendrimer backbone [103,104], contrary to the neo-glycoproteins in the present study. Moreover, calixarenes multivalently presenting lactose increased the inhibitory potency towards Gal-3 binding to ASF by a factor of 12.5 reaching an IC_50_ of 200 µM [89]. Multivalent LacNAc bearing calix[4/6]arenes with 2/3' substitutions in the LacNAc core showed IC_50_ values up to 0.15 µM for Gal-3 binding to ASF, which were about 1500 times lower compared to monovalent species [90]. Hence, multivalent presented LacNAc increases Gal-3 binding more than multivalent lactose. In the present study, multivalent presentation of tetrasaccharides on BSA yields lower IC_50_ values up to 0.04 µM indicating a large benefit using tetrasaccharides instead of disaccharides.

In conclusion, we could prove inhibitory potency for Gal-3 binding to ASF applying the presented neo-glycoproteins. For modification densities of at least 18 glycans per BSA, up to 15 times higher potencies in relation to one binding site are recorded. We suggest that IC_50_ values can even be reduced in assays using lower Gal-3 concentration, e.g., *in vivo* assays, as we could show up to 100 times increased Gal-3 binding at concentrations below 0.05 µM (Figure 5).

## 3. Experimental Section

### 3.1. Production of Recombinant Enzymes

Enzymes were expressed and purified as described previously [65,67,76,105]. Briefly, the fusion protein of human β1,4-galactosyltransferase-1 (His_6_-Propeptide-catβ4GalT-1, β4GalT) and the Y284L mutant (β4GalTY284L) were expressed in *E. coli* Shuffle T7 Express (DE3) (NEB, Frankfurt/Main, Germany). The β1,3-*N*-acetylglucosaminyltransferase from *Helicobacter pylori* (β3GlcNAcT) and UDP-Glc 4′-epimerase from *Campylobacter jejuni*, both fused to maltose binding protein, were expressed in *E. coli* BL21 (DE3) (Novagen/Merck, Darmstadt, Germany). TB medium with 0.5 mM IPTG was used for induction of expression. After 24 h, cells were harvested by centrifugation and appropriate cell mass was sonicated two times for 30 s for follow-up affinity chromatography. Recombinant MBP-tagged proteins were purified by amylose affinity chromatography using MBPTrap^TM^ HP 5 mL column (GE Healthcare, Munich, Germany) and His_6_-tagged proteins by immobilized metal-ion affinity chromatography (IMAC) using HisTrap^TM^ HP 5 mL column (GE Healthcare). Purification was carried out according to manufacturer’s instructions. After column equilibration bacterial crude extracts were loaded onto the column at a flow rate of 1 mL/min and washed until baseline signal was reached. Target proteins were eluted by application of elution buffer containing 10 mM maltose or 500 mM imidazole. The buffer for β4GalTY284L was subsequently changed to 0.1 M MOPS buffer (pH 6.8) including 20% (v/v) glycerol.

### 3.2. Chemo-Enzymatic Synthesis of Glycans (**4** and **5**)

Compound **1** (GlcNAc-linker-NH_2_-*t*Boc) was synthesized as described [105] and served as starting material for synthesis of **4** (Galβ1, 4GlcNAcβ1, 3Galβ1, 4GlcNAcβ1-linker-NH_2_-*t*Boc, LacNAc-LacNAc-linker-NH_2_-*t*Boc) and **5** (GalNAcβ1,4GlcNAcβ1,3Galβ1,4GlcNAcβ1-linker-NH_2_-*t*Boc, LacDiNAc-LacNAc-linker-NH_2_-*t*Boc). **1** was elongated sequentially using β4GalT adding galactose resulting in **2** (Galβ1,4GlcNAcβ1-linker-NH_2_-*t*Boc) [67] and β3GlcNAcT producing **3** (GlcNAcβ1, 3Galβ1, 4GlcNAcβ1-linker-NH_2_-*t*Boc) [105]. Either galactose using β4GalT or GalNAc using β4GalTY284L with *in-situ* production of UDP-GalNAc from UDP-GlcNAc using UDP-Glc 4'-epimerase were added to **3** yielding **4** and **5**, respectively. The enzymatic synthesis of compound **4** was carried out as described for compound **2**. To synthesize **5** compound **3** (5 mM) was mixed with 15 mM UDP-GlcNAc (Roche, Mannheim, Germany) in reaction buffer (100 mM MOPS-NaOH, pH 6, with 25 mM KCl, 2 mM MnCl_2_, 10 mU/mL alkaline phosphatase), 75 mU/mL β4GalTY284L and 7 U/mL U UDP-Glc 4'-epimerase. Reaction was monitored via HPLC (LiChrospher 100 RP 18-5µ, 15% (v/v) acetonitrile, 0.1% (v/v) formic acid or Multospher APS-HP 5µ, 5 mM ammonium acetate (pH 4.3), 80% (v/v) acetonitrile). To stop the reaction, enzymes were removed via filtration (VivaSpin^®^20, Sartorius, Göttingen, Germany) and products were purified by solid phase extraction with Sep-Pak^®^ Vac C18 1cc columns (Waters GmbH, Eschborn, Germany) [105] followed by mass analysis by LC-ESI-MS.

### 3.3. Synthesis of Compounds **9** and **10**

Deprotection of **4** and **5** was carried out under acidic conditions (1 M HCl) resulting in **6** and **7** followed by neutralization using Dowex^®^ 66 free base (Sigma-Aldrich, Taufkirchen, Germany). Compounds **6** and **7** were dissolved in 50% aqueous ethanol (35 mM HEPES, pH 7.0) containing 4 equivalents of **8** (squaric acid diethyl ester, Sigma-Aldrich) and of triethylamine [72]. Then, 27.24 mg or 28.38 mg (28.67 µmol) of **6** and **7**, respectively, were mixed with 19.51 mg (114.68 µmol) of **8** and trimethylamine (11.6 mg). Reaction mixture was incubated at room temperature overnight while gently shaking. When TLC (Alugram^®^ Xtra SIL G/UV254, Macherey-Nagel, Düren, Germany, n‑butanol/ethanol/water/15% aqueous ammonia, 5:10:8:4) showed disappearance of oligosaccharide starting material, the solvent was evaporated and products **9** and **10**, respectively, were purified by preparative HPLC (Eurospher 100-10 C18, 15% (v/v) acetonitrile). The correct masses were proven by LC-ESI-MS and concentration was determined via HPLC (LiChrospher 100 RP 18-5µ, 15% (v/v) acetonitrile, 0.1% (v/v) formic acid) using calibration curve of GlcNAc conjugated to **8**.

### 3.4. Synthesis of Neo-Glycoproteins Based on BSA

Prior to BSA modification, active charcoal delipidation was carried out for removal of lipid contaminations as described [106], because commercial preparations of BSA may contain fatty compounds. Coupling reaction to BSA (Carl Roth, Karlsruhe, Germany) was carried out under slightly basic conditions using sodium tetraborate buffer (50 mM, pH 9.0). Compounds **9** and **10** dissolved in defined volume of water were applied in varying molar ratios compared to lysine residues of BSA (from 1:60 to 1.5:1). Reaction mixtures were incubated for up to 136 h at room temperature while gently shaking. The products **11a–k** and **12a–k** were purified via filtration to remove unbound glycans and analyzed regarding BSA lysine residue modification as described below.

### 3.5. Analysis of Neo-Glycoproteins

Determination of free lysine residues (ε-amino groups) in proteins was carried out using 2,4,6‑trinitrobenzene sulfonic acid (TNBSA, Sigma-Aldrich) as detecting agent. The TNBSA assay was performed as described [80] but optimized for our application. Protein samples were diluted in 50 mM sodium tetraborate buffer (pH 9.0) to yield a concentration of 12.5 µM and 50 µL were mixed with equal volume of 7.5 mM TNBSA. After incubation at room temperature for 15 min, absorbance at 420 nm was measured in microplate reader (SPECTRAmax Plus, Molecular Devices, Ismaning, Germany). Standard curves were generated using lysine hydrochloride and crude BSA as standard. Protein amounts were measured by Bradford assay using calibration with unmodified BSA.

The modification of BSA with glycans resulted in increasing molecular weights that were verified by SDS-PAGE. SDS-PAGE was done using 8% gels and constant current of 25 mA.

### 3.6. Expression and Purification of Recombinant Galectins

Human His_6_Gal-1C2S and human His_6_Gal-3 were expressed and purified as described elsewhere [66,107] except that we used *E. coli* Rosetta (DE3) pLysS (Novagen/Merck, Darmstadt, Germany) for expression. The purification via IMAC was performed as mentioned above. His_6_Gal-1C2S and His_6_Gal-3 were stored at 4 °C in phosphate buffered saline containing 2 mM EDTA (EPBS, pH 7.5) including 10% (v/v) glycerol for His_6_Gal-1C2S. Protein amounts were measured by Bradford assay using calibration with BSA.

### 3.7. Galectin Binding Assays on Neo-Glycoproteins

The potential of neo-glycoproteins acting as ligands for galectins was evaluated in an ELISA-type assay [66]. The neo-glycoproteins **11a–i** and **12a–i** as well as unmodified BSA (50 µL of 0.1 µM protein in PBS pH 7.5) were immobilized in microtiter plates (MaxiSorp, Nunc, Wiesbaden, Germany) overnight. Residual binding sites were blocked with 2% delipidated BSA in PBS followed by 1 h incubation of galectin in EPBS. Anti-His_6_-peroxidase (Roche, Mannheim, Germany, 1:2000 in PBS) was used to detect bound galectin that subsequently converted OPD substrate (o-phenylenediamine, Dako, Hamburg, Germany) with read-out at 492 nm. Between the different incubation steps wells were washed three times with PBS-Tween (0.05% (v/v)). Measured data were fitted and K_d_ values calculated using Sigma Plot (Systat software GmbH, Erkrath, Germany). Significance of the data was investigated by independent two sample t-test with *p* < 0.002.

### 3.8. Inhibition of Galectin Binding with Neo-Glycoproteins

Neo-glycoproteins were used as inhibitors of Gal-3 to ASF binding in competitive inhibition assays performed as described [66]. The standard glycoprotein ASF (Sigma-Aldrich) was immobilized in microtiter plates as described above for neo-glycoproteins. After blocking, 5 µM Gal-3 and varying concentration of **11j**, **11k**, **11g** and **12j**, **12k**, **12g** were simultaneously incubated for 1 h followed by detection of bound Gal-3 as described above. Measured data were fitted and IC_50_ values calculated using Sigma Plot software (Systat software GmbH).

## 4. Conclusions

We here demonstrate the efficient synthesis of high-affinity ligands for human Gal-3. We show that LacNAc-LacNAc or LacDiNAc-LacNAc conjugation to BSA is tunable using squaric acid diethyl ester as linker. We report here for the first time on selective Gal-3 binding to multivalent neo-glycoproteins revealing and quantifying the influence of defined multivalency. The highest binding enhancement by a factor of about 100 was observed with LacDiNAc-LacNAc conjugated BSA modified with at least 21 glycans at a Gal-3 concentration of 5 nM. The efficient and selective binding of such neo-glycoproteins at serum level concentrations of Gal-3 may have high impact in anti-cancer therapy. The here presented multivalent neo-glycoproteins can be further loaded with cytotoxic compounds and labeled with fluorescent dyes enabling tumor diagnostic by molecular imaging and tumor therapy. In summary, our tailor-made neo-glycoproteins are suitable candidates for targeting Gal-3 in cancer related biomedical research.

## Supplementary Files

Supplementary File 1

## References

[1] Varki A. (1993). Biological roles of oligosaccharides: All of the theories are correct. Glycobiology.

[2] Zhao Y.-Y., Takahashi M., Gu J.-G., Miyoshi E., Matsumoto A., Kitazume S., Taniguchi N. (2008). Functional roles of *N*-glycans in cell signaling and cell adhesion in cancer. Cancer Sci..

[3] Boscher C., Dennis J.W., Nabi I.R. (2011). Glycosylation, galectins and cellular signaling. Curr. Opin. Cell Biol..

[4] Fred Brewer C. (2002). Binding and cross-linking properties of galectins. BBA Gen. Subj..

[5] Funasaka T., Raz A., Nangia-Makker P. (2014). Galectin-3 in angiogenesis and metastasis. Glycobiology.

[6] Lau K.S., Dennis J.W. (2008). *N*-glycans in cancer progression. Glycobiology.

[7] Noorjahan P. (2012). Role of galectins in wound healing. Galectins and Disease Implications for Targeted Therapeutics.

[8] Schattner M. (2014). Platelets and galectins. Ann. Transl. Med..

[9] Lundquist J.J., Toone E.J. (2002). The cluster glycoside effect. Chem. Rev..

[10] Kiessling L.L., Young T., Gruber T.D., Mortell K.H., Fraser-Reid B., Tatsuta K., Thiem J. (2008). Multivalency in protein-carbohydrate recognition. Glycoscience.

[11] Pieters R.J. (2009). Maximising multivalency effects in protein-carbohydrate interactions. Org. Biomol. Chem..

[12] Pieters R.J., Arnusch C.J., Breukink E. (2009). Membrane permeabilization by multivalent anti-microbial peptides. Protein Pept. Lett..

[13] Bernardi A., Jimenez-Barbero J., Casnati A., de Castro C., Darbre T., Fieschi F., Finne J., Funken H., Jaeger K.-E., Lahmann M. (2013). Multivalent glycoconjugates as anti-pathogenic agents. Chem. Soc. Rev..

[14] Peri F. (2013). Clustered carbohydrates in synthetic vaccines. Chem. Soc. Rev..

[15] Branson T.R., Turnbull W.B. (2013). Bacterial toxin inhibitors based on multivalent scaffolds. Chem. Soc. Rev..

[16] Chabre Y.M., Roy R. (2013). Multivalent glycoconjugate syntheses and applications using aromatic scaffolds. Chem. Soc. Rev..

[17] Marradi M., Chiodo F., Garcia I., Penades S. (2013). Glyconanoparticles as multifunctional and multimodal carbohydrate systems. Chem. Soc. Rev..

[18] Martinez A., Ortiz Mellet C., Garcia Fernandez J.M. (2013). Cyclodextrin-based multivalent glycodisplays: Covalent and supramolecular conjugates to assess carbohydrate-protein interactions. Chem. Soc. Rev..

[19] Hatano K., Matsuoka K., Terunuma D. (2013). Carbosilane glycodendrimers. Chem. Soc. Rev..

[20] Chen Y., Star A., Vidal S. (2013). Sweet carbon nanostructures: Carbohydrate conjugates with carbon nanotubes and graphene and their applications. Chem. Soc. Rev..

[21] Kennedy D.C., Grünstein D., Lai C.-H., Seeberger P.H. (2013). Glycosylated nanoscale surfaces: Preparation and applications in medicine and molecular biology. Chem. Eur. J..

[22] Sansone F., Casnati A. (2013). Multivalent glycocalixarenes for recognition of biological macromolecules: Glycocalyx mimics capable of multitasking. Chem. Soc. Rev..

[23] Cecioni S., Imberty A., Vidal S. (2014). Glycomimetics *versus* multivalent glycoconjugates for the design of high affinity lectin ligands. Chem. Rev..

[24] Bojarová P., Rosencrantz R.R., Elling L., Křen V. (2013). Enzymatic glycosylation of multivalent scaffolds. Chem. Soc. Rev..

[25] Ercolani G., Schiaffino L. (2011). Allosteric, chelate, and interannular cooperativity: A mise AU point. Angew. Chem. Int. Ed..

[26] Hunter C.A., Anderson H.L. (2009). What is cooperativity?. Angew. Chem. Int. Ed..

[27] Maierhofer C., Rohmer K., Wittmann V. (2007). Probing multivalent carbohydrate-lectin interactions by an enzyme-linked lectin assay employing covalently immobilized carbohydrates. Bioorg. Med. Chem..

[28] Lee R., Lee Y. (2000). Affinity enhancement by multivalent lectin-carbohydrate interaction. Glycoconj. J..

[29] Profit A.A., Lee T.R., Lawrence D.S. (1999). Bivalent inhibitors of protein tyrosine kinases. J. Am. Chem. Soc..

[30] Schaschke N., Matschiner G., Zettl F., Marquardt U., Bergner A., Bode W., Sommerhoff C.P., Moroder L. (2001). Bivalent inhibition of human β-tryptase. Chem. Biol..

[31] Rao J., Lahiri J., Isaacs L., Weis R.M., Whitesides G.M. (1998). A trivalent system from vancomycin·d-Ala-d-Ala with higher affinity than avidin·biotin. Science.

[32] Blackmore P.F., Eisoldt S. (1999). The neoglycoprotein mannose-bovine serum albumin, but not progesterone, activates T-type calcium channels in human spermatozoa. Mol. Hum. Reprod..

[33] Oh Y.S., Ahn H.S., Gye M.C. (2013). Fucosyl neoglycoprotein binds to mouse epididymal spermatozoa and inhibits sperm binding to the egg zona pellucida. Andrologia.

[34] Huang B.X., Kim H.-Y., Dass C. (2004). Probing three-dimensional structure of bovine serum albumin by chemical cross-linking and mass spectrometry. J. Am. Soc. Mass Spectrom..

[35] Luyai A., Lasanajak Y., Smith D.F., Cummings R.D., Song X. (2009). Facile preparation of fluorescent neoglycoproteins using p-nitrophenyl anthranilate as a heterobifunctional linker. Bioconjug. Chem..

[36] Unverzagt C., André S., Seifert J., Kojima S., Fink C., Srikrishna G., Freeze H., Kayser K., Gabius H.-J. (2002). Structure-activity profiles of complex biantennary glycans with core fucosylation and with/without additional α2,3/α2,6 sialylation:  Synthesis of neoglycoproteins and their properties in lectin assays, cell binding, and organ uptake. J. Med. Chem..

[37] Gabius H.J., Bardosi A. (1991). Neoglycoproteins as tools in glycohistochemistry. Prog. Histochem. Cytochem..

[38] Stowell C.P., Lee Y.C., Tipson R.S., Derek H. (1980). Neoglycoproteins the preparation and application of synthetic glycoproteins. Advances in Carbohydrate Chemistry and Biochemistry.

[39] Kerekgyarto M., Fekete A., Szurmai Z., Kerekgyarto J., Takacs L., Kurucz I., Guttman A. (2013). Neoglycoproteins as carbohydrate antigens: Synthesis, analysis, and polyclonal antibody response. Electrophoresis.

[40] Barondes S.H., Cooper D.N., Gitt M.A., Leffler H. (1994). Galectins. Structure and function of a large family of animal lectins. J. Biol. Chem..

[41] Leffler H., Carlsson S., Hedlund M., Qian Y., Poirier F. (2002). Introduction to galectins. Glycoconj. J..

[42] Griffioen A.W., Thijssen V.L. (2014). Galectins in tumor angiogenesis. Ann. Transl. Med..

[43] Rabinovich G.A., van Kooyk Y., Cobb B.A. (2012). Glycobiology of immune responses. Ann. NY Acad. Sci..

[44] Liu F.-T., Rabinovich G.A. (2005). Galectins as modulators of tumour progression. Nat. Rev. Cancer.

[45] D’Haene N., Maris C., Rorive S., Decaestecker C., le Mercier M., Salmon I. (2014). Galectins and neovascularization in central nervous system tumors. Glycobiology.

[46] Compagno D., Gentilini L.D., Jaworski F.M., Pérez I.G., Contrufo G., Laderach D.J. (2014). Glycans and galectins in prostate cancer biology, angiogenesis and metastasis. Glycobiology.

[47] Le Mercier M., Fortin S., Mathieu V., Kiss R., Lefranc F. (2010). Galectins and gliomas. Brain Pathol..

[48] Nakahara S., Oka N., Raz A. (2005). On the role of galectin-3 in cancer apoptosis. Apoptosis.

[49] Dumic J., Dabelic S., Flögel M. (2006). Galectin-3: An open-ended story. BBA Gen. Subj..

[50] Pugliese G., Iacobini C., Pesce C.M., Menini S. (2015). Galectin-3: An emerging all-out player in metabolic disorders and their complications. Glycobiology.

[51] Hakon L., Ulf J.N. (2012). Low-molecular weight inhibitors of galectins. Galectins and Disease Implications for Targeted Therapeutics.

[52] Stowell S.R., Arthur C.M., Mehta P., Slanina K.A., Blixt O., Leffler H., Smith D.F., Cummings R.D. (2008). Galectin-1,-2, and-3 exhibit differential recognition of sialylated glycans and blood group antigens. J. Biol. Chem..

[53] Téllez-Sanz R., Garcia-Fuentes L., Vargas-Berenguel A. (2013). Human galectin-3 selective and high affinity inhibitors. Present state and future perspectives. Curr. Med. Chem..

[54] Ahmad N., Gabius H.J., Andre S., Kaltner H., Sabesan S., Roy R., Liu B.C., Macaluso F., Brewer C.F. (2004). Galectin-3 precipitates as a pentamer with synthetic multivalent carbohydrates and forms heterogeneous cross-linked complexes. J. Biol. Chem..

[55] Lepur A., Salomonsson E., Nilsson U.J., Leffler H. (2012). Ligand induced galectin-3 protein self-association. J. Biol. Chem..

[56] Dam T.K., Gabius H.-J., André S., Kaltner H., Lensch M., Brewer C.F. (2005). Galectins bind to the multivalent glycoprotein asialofetuin with enhanced affinities and a gradient of decreasing binding constants. Biochemistry.

[57] Goodman C.K., Wolfenden M.L., Nangia-Makker P., Michel A.K., Raz A., Cloninger M.J. (2014). Multivalent scaffolds induce galectin-3 aggregation into nanoparticles. Beilstein J. Org. Chem..

[58] Yang Y., Xue X.C., Jin X.F., Wang L.J., Sha Y.L., Li Z.J. (2012). Synthesis of multivalent n-acetyl lactosamine modified quantum dots for the study of carbohydrate and galectin-3 interactions. Tetrahedron.

[59] Wolfenden M., Cousin J., Nangia-Makker P., Raz A., Cloninger M. (2015). Glycodendrimers and modified elisas: Tools to elucidate multivalent interactions of galectins 1 and 3. Molecules.

[60] Van den Berg T.K., Honing H., Franke N., van Remoortere A., Schiphorst W., Liu F.T., Deelder A.M., Cummings R.D., Hokke C.H., van Die I. (2004). Lacdinac-glycans constitute a parasite pattern for galectin-3-mediated immune recognition. J. Immunol..

[61] Hirano K., Matsuda A., Shirai T., Furukawa K. Expression of lacdinac group on *N*-glycans among human tumors is complex. Biomed. Res. Int..

[62] Do K.-Y., Do S., Cummings R.D. (1997). Differential expression of lacdinac sequences (GalNAcβ1-4GlcNAc-R) in glycoproteins synthesized by chinese hamster ovary and human 293 cells. Glycobiology.

[63] Kenny D.T., Skoog E.C., Lindén S.K., Struwe W.B., Rudd P.M., Karlsson N.G. (2012). Presence of terminal *N*-acetylgalactosamineβ1-4*N*-acetylglucosamine residues on *O*-linked oligosaccharides from gastric MUC5AC: Involvement in helicobacter pylori colonization?. Glycobiology.

[64] Rossez Y., Gosset P., Boneca I.G., Magalhaes A., Ecobichon C., Reis C.A., Cieniewski-Bernard C., Joncquel Chevalier Curt M., Leonard R., Maes E. (2014). The lacdinac-specific adhesin laba mediates adhesion of helicobacter pylori to human gastric mucosa. J. Infect. Dis..

[65] Šimonová A., Kupper C.E., Böcker S., Müller A., Hofbauerová K., Pelantová H., Elling L., Křen V., Bojarová P. (2014). Chemo-enzymatic synthesis of lacdinac dimers of varying length as novel galectin ligands. J. Mol. Catal. B Enzym..

[66] Kupper C.E., Böcker S., Liu H.L., Adamzyk C., van de Kamp J., Recker T., Lethaus B., Jahnen-Dechent W., Neuss S., Müller-Newen G. (2013). Fluorescent SNAP-Tag galectin fusion proteins as novel tools in glycobiology. Curr. Pharm. Des..

[67] Rech C., Rosencrantz R.R., Křenek K., Pelantová H., Bojarová P., Römer C.E., Hanisch F.-G., Křen V., Elling L. (2011). Combinatorial one-pot synthesis of poly-*N*-acetyllactosamine oligosaccharides with leloir-glycosyltransferases. Adv. Synth. Catal..

[68] Kamath V.P., Diedrich P., Hindsgaul O. (1996). Use of diethyl squarate for the coupling of oligosaccharide amines to carrier proteins and characterization of the resulting neoglycoproteins by MALDI-TOF mass spectrometry. Glycoconj. J..

[69] Tietze L.F., Arlt M., Beller M., gl üsenkamp K.-H., Jähde E., Rajewsky M.F. (1991). Anticancer agents, 15. Squaric acid diethyl ester: A new coupling reagent for the formation of drug biopolymer conjugates. Synthesis of squaric acid ester amides and diamides. Chem. Ber..

[70] Tietze L.F., Schroeter C., Gabius S., Brinck U., Goerlach-Graw A., Gabius H.J. (1991). Conjugation of p-aminophenyl glycosides with squaric acid diester to a carrier protein and the use of the neoglycoprotein in the histochemical detection of lectins. Bioconjug. Chem..

[71] Wang J.W., Asnani A., Auzanneau F.I. (2010). Synthesis of a BSA-Le^x^ glycoconjugate and recognition of Le^x^ analogues by the anti-Le^x^ monoclonal antibody sh1: The identification of a non-cross reactive analogue. Bioorg. Med. Chem..

[72] Wurm F.R., Klok H.A. (2013). Be squared: Expanding the horizon of squaric acid-mediated conjugations. Chem. Soc. Rev..

[73] Komarova B.S., Orekhova M.V., Tsvetkov Y.E., Beau R., Aimanianda V., Latge J.P., Nifantiev N.E. (2015). Synthesis of a pentasaccharide and neoglycoconjugates related to fungal alpha-(1→3)-glucan and their use in the generation of antibodies to trace aspergillus fumigatus cell wall. Chemistry.

[74] Jahouh F., Hou S.J., Kovac P., Banoub J.H. (2012). Determination of glycation sites by tandem mass spectrometry in a synthetic lactose-bovine serum albumin conjugate, a vaccine model prepared by dialkyl squarate chemistry. Rapid. Commun. Mass Spectrom..

[75] Jahouh F., Xu P., Vann W.F., Kovac P., Banoub J.H. (2013). Mapping the glycation sites in the neoglycoconjugate from hexasaccharide antigen of vibrio cholerae, serotype ogawa and the recombinant tetanus toxin C-fragment carrier. J. Mass Spectrom..

[76] Sauerzapfe B., Namdjou D.J., Schumacher T., Linden N., Křenek K., Křen V., Elling L. (2008). Characterization of recombinant fusion constructs of human β1,4-galactosyltransferase 1 and the lipase pre-propeptide from staphylococcus hyicus. J. Mol. Catal. B Enzym..

[77] Logan S.M., Altman E., Mykytczuk O., Brisson J.R., Chandan V., Schur M.J., St Michael F., Masson A., Leclerc S., Hiratsuka K. (2005). Novel biosynthetic functions of lipopolysaccharide rfaj homologs from helicobacter pylori. Glycobiology.

[78] Kupper C.E., Rosencrantz R.R., Henssen B., Pelantová H., Thönes S., Drozdova A., Křen V., Elling L. (2012). Chemo-enzymatic modification of poly-*N*-acetyllactosamine (LacNAc) oligomers and *N,N*-diacetyllactosamine (LacDINAc) based on galactose oxidase treatment. Beilstein J. Org. Chem..

[79] Hou S.J., Saksena R., Kovac P. (2008). Preparation of glycoconjugates by dialkyl squarate chemistry revisited. Carbohydr. Res..

[80] Cayot P., Tainturier G. (1997). The quantification of protein amino groups by the trinitrobenzenesulfonic acid method: A reexamination. Anal. Biochem..

[81] Roth Z., Yehezkel G., Khalaila I. (2012). Identification and quantification of protein glycosylation. Int. J. Carbohydr. Chem..

[82] Ahmad N., Gabius H.J., Kaltner H., Andre S., Kuwabara I., Liu F.T., Oscarson S., Norberg T., Brewer C.F. (2002). Thermodynamic binding studies of cell surface carbohydrate epitopes to galectins-1,-3, and-7: Evidence for differential binding specificities. Can. J. Chem..

[83] Rapoport E.M., Andre S., Kurmyshkina O.V., Pochechueva T.V., Severov V.V., Pazynina G.V., Gabius H.J., Bovin N.V. (2008). Galectin-loaded cells as a platform for the profiling of lectin specificity by fluorescent neoglycoconjugates: A case study on galectins-1 and-3 and the impact of assay setting. Glycobiology.

[84] Stowell S.R., Dias-Baruffi M., Penttila L., Renkonen O., Nyame A.K., Cummings R.D. (2004). Human galectin-1 recognition of poly-N-acetyllactosamine and chimeric polysaccharides. Glycobiology.

[85] Leppänen A., Stowell S., Blixt O., Cummings R.D. (2005). Dimeric galectin-1 binds with high affinity to alpha 2,3-sialylated and non-sialylated terminal *N*-acetyllactosamine units on surface-bound extended glycans. J. Biol. Chem..

[86] Di Virgilio S., Glushka J., Moremen K., Pierce M. (1999). Enzymatic synthesis of natural and C-13 enriched linear poly-N-acetyllactosamines as ligands for galectin-1. Glycobiology.

[87] Qun Z., Cummings R.D. (1993). L-14 lectin recognition of laminin and its promotion of *in vitro* cell adhesion. Arch. Biochem. Biophys..

[88] Wang H., Huang W., Orwenyo J., Banerjee A., Vasta G.R., Wang L.X. (2013). Design and synthesis of glycoprotein-based multivalent glyco-ligands for influenza hemagglutinin and human galectin-3. Bioorg. Med. Chem..

[89] André S., Sansone F., Kaltner H., Casnati A., Kopitz J., Gabius H.J., Ungaro R. (2008). Calix N arene-based glycoclusters: Bioactivity of thiourea-linked galactose/lactose moieties as inhibitors of binding of medically relevant lectins to a glycoprotein and cell-surface glycoconjugates and selectivity among human adhesion/growth-regulatory galectins. ChemBioChem.

[90] André S., Grandjean C., Gautier F.M., Bernardi S., Sansone F., Gabius H.J., Ungaro R. (2011). Combining carbohydrate substitutions at bioinspired positions with multivalent presentation towards optimising lectin inhibitors: Case study with calixarenes. Chem. Commun..

[91] André S., Pieters R.J., Vrasidas I., Kaltner H., Kuwabara I., Liu F.T., Liskamp R.M., Gabius H.J. (2001). Wedgelike glycodendrimers as inhibitors of binding of mammalian galectins to glycoproteins, lactose maxiclusters, and cell surface glycoconjugates. ChemBioChem.

[92] Song L., Tang J.W., Owusu L., Sun M.Z., Wu J., Zhang J. (2014). Galectin-3 in cancer. Clin. Chim. Acta.

[93] Saussez S., Lorfevre F., Lequeux T., Laurent G., Chantrain G., Vertongen F., Toubeau G.R., Decaestecker C., Kiss R. (2008). The determination of the levels of circulating galectin-1 and -3 in HNSCC patients could be used to monitor tumor progression and/or responses to therapy. Oral. Oncol..

[94] Sakaki M., Oka N., Nakanishi R., Yamaguchi K., Fukumori T., Kanayama H.O. (2008). Serum level of galectin-3 in human bladder cancer. J. Med. Invest..

[95] Iurisci I., Tinari N., Natoli C., Angelucci D., Cianchetti E., Iacobelli S. (2000). Concentrations of galectin-3 in the sera of normal controls and cancer patients. Clin. Cancer Res..

[96] Johnson K.D., Glinskii O.V., Mossine V.V., Turk J.R., Mawhinney T.P., Anthony D.C., Henry C.J., Huxley V.H., Glinsky G.V., Pienta K.J. (2007). Galectin-3 as a potential therapeutic target in tumors arising from malignant endothelia. Neoplasia.

[97] Sörme P., Arnoux P., Kahl-Knutsson B., Leffler H., Rini J.M., Nilsson U.J. (2005). Structural and thermodynamic studies on cation-Pi interactions in lectin-ligand complexes: High-affinity galectin-3 inhibitors through fine-tuning of an arginine-arene interaction. J. Am. Chem. Soc..

[98] Nangia-Makker P., Hogan V., Honjo Y., Baccarini S., Tait L., Bresalier R., Raz A. (2002). Inhibition of human cancer cell growth and metastasis in nude mice by oral intake of modified citrus pectin. J. Natl. Cancer Inst..

[99] Iurisci I., Cumashi A., Sherman A.A., Tsvetkov Y.E., Tinari N., Piccolo E., D’Egidio M., Adamo V., Natoli C., Rabinovich G.A. (2009). Synthetic inhibitors of galectin-1 and -3 selectively modulate homotypic cell aggregation and tumor cell apoptosis. Anticancer Res..

[100] Michel A.K., Nangia-Makker P., Raz A., Cloninger M.J. (2014). Lactose-functionalized dendrimers arbitrate the interaction of galectin-3/muc1 mediated cancer cellular aggregation. ChemBioChem.

[101] Prasanphanich N.S., Song X., Heimburg-Molinaro J., Luyai A.E., Lasanajak Y., Cutler C.E., Smith D.F., Cummings R.D. (2015). Intact reducing glycan promotes the specific immune response to lacto-n-neotetraose-bsa neoglycoconjugates. Bioconjug. Chem..

[102] Mencke A.J., Wold F. (1982). Neoglycoproteins. Preparation and *in vivo* clearance of serum albumin derivatives containing ovalbumin oligosaccharides. J. Biol. Chem..

[103] Vrasidas I., Andre S., Valentini P., Bock C., Lensch M., Kaltner H., Liskamp R.M., Gabius H.J., Pieters R.J. (2003). Rigidified multivalent lactose molecules and their interactions with mammalian galectins: A route to selective inhibitors. Org. Biomol. Chem..

[104] Parera Pera N., Branderhorst H.M., Kooij R., Maierhofer C., van der Kaaden M., Liskamp R.M., Wittmann V., Ruijtenbeek R., Pieters R.J. (2010). Rapid screening of lectins for multivalency effects with a glycodendrimer microarray. ChemBioChem.

[105] Sauerzapfe B., Krenek K., Schmiedel J., Wakarchuk W.W., Pelantova H., Kren V., Elling L. (2009). Chemo-enzymatic synthesis of poly-N-acetyllactosamine (poly-LacNAc) structures and their characterization for CGL2-galectin-mediated binding of ecm glycoproteins to biomaterial surfaces. Glycoconj. J..

[106] Chen R.F. (1967). Removal of fatty acids from serum albumin by charcoal treatment. J. Biol. Chem..

[107] Witten K.G., Rech C., Eckert T., Charrak S., Richtering W., Elling L., Simon U. (2011). Glyco-DNA-gold nanoparticles: Lectin-mediated assembly and dual-stimuli response. Small.

