# Supplementary material for: Galectin Binding to Neo-Glycoproteins: LacDiNAc Conjugated BSA as Ligand for Human Galectin-3
Source: Biomolecules. 2015 Jul 24;5(3):1671–96. doi: 10.3390/biom5031671 (PMC4598770; doi:10.3390/biom5031671)
Supplement: Supplementary File 1 [file biomolecules-05-01671-s001.pdf]

Supplementary Information

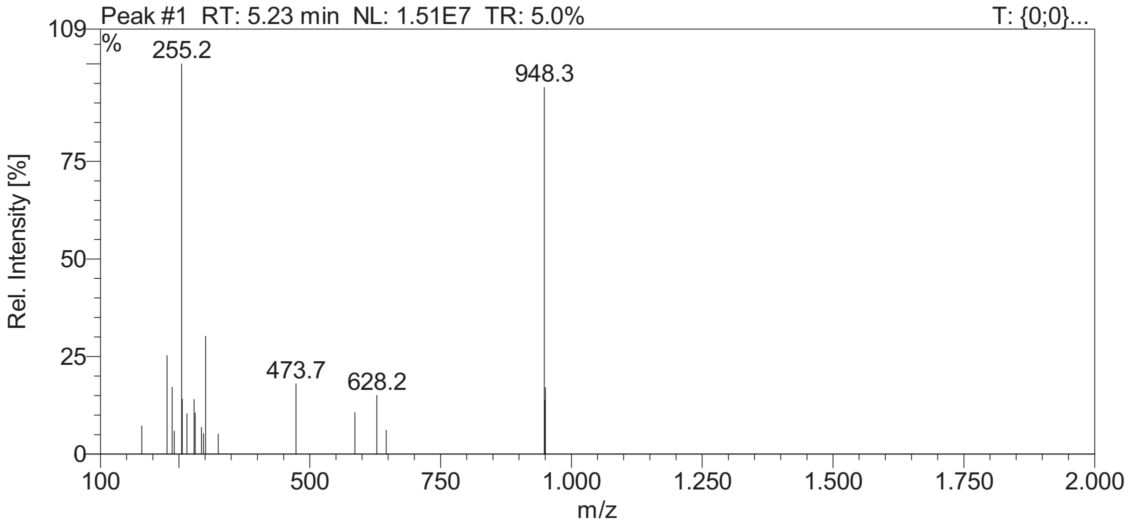

(**a**)

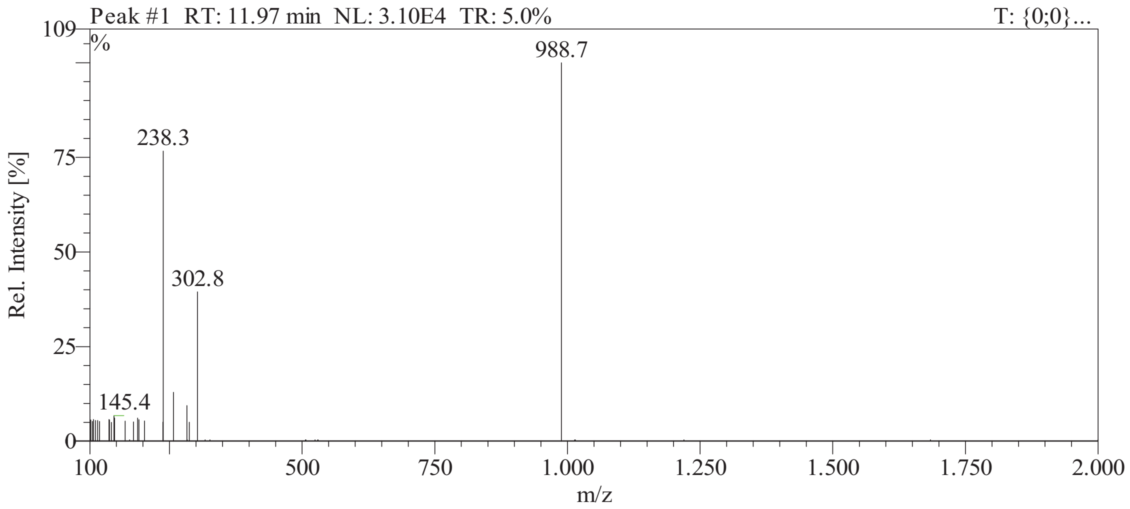

(**b**)

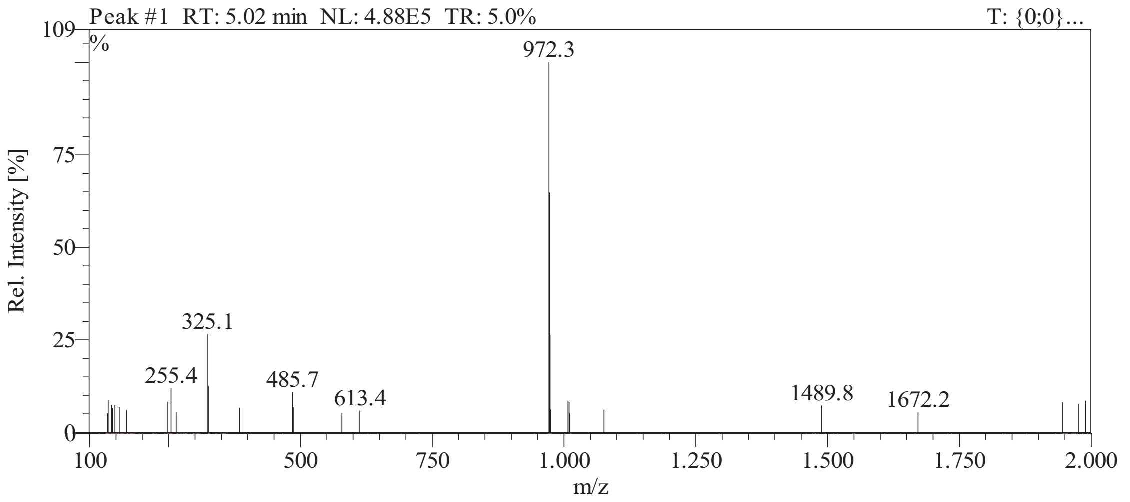

(**c**)

Figure S1. *Cont.*

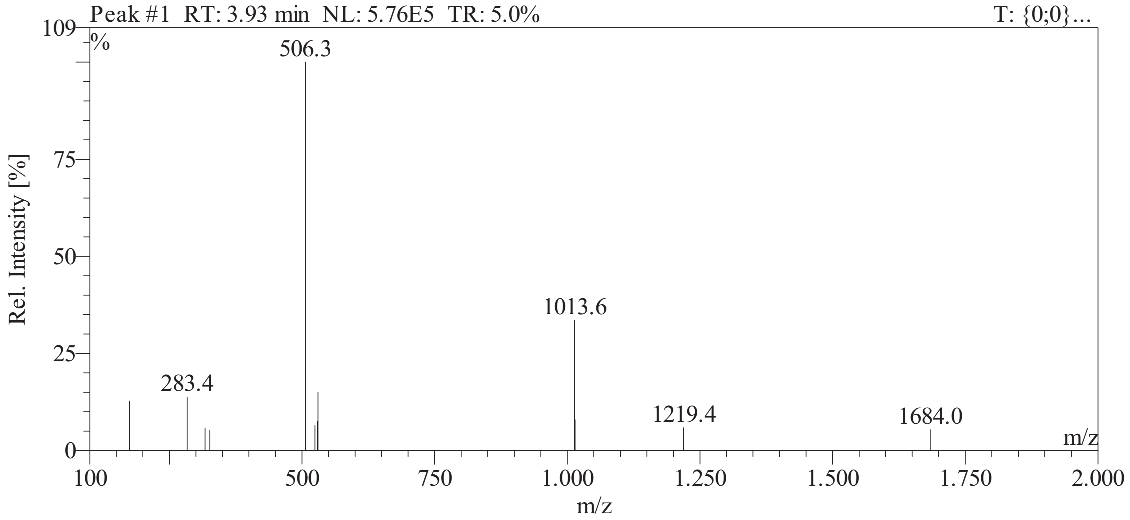

(**d**)

Figure S1. MS spectra of substances **4** (**a**), **5** (**b**), **9** (**c**) and **10** (**d**). Via LC-MS using electrospray ionization $[M-H]^-$ ions were analyzed using quadrupole mass analyzer. **4** $[M-H]^-$ = 949.97 *m*/*z*, **5** $[M\text{-}H]^-$ = 990.01 *m*/*z*, **9** $[M-H]^-$ = 973.95 *m*/*z* and **10** $[M-H]^-$ = 1013.99 *m*/*z* ($[M-2H]^{2-}$ = 506.3 *m*/*z*) could be verified ((**a**)–(**d**)).

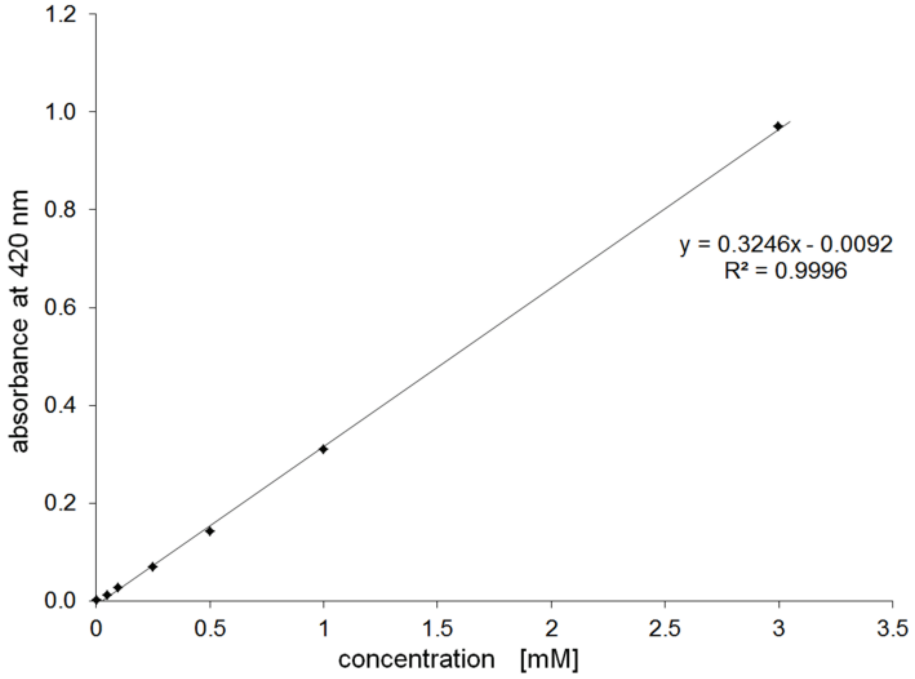

(**a**)

Figure S2. *Cont.*

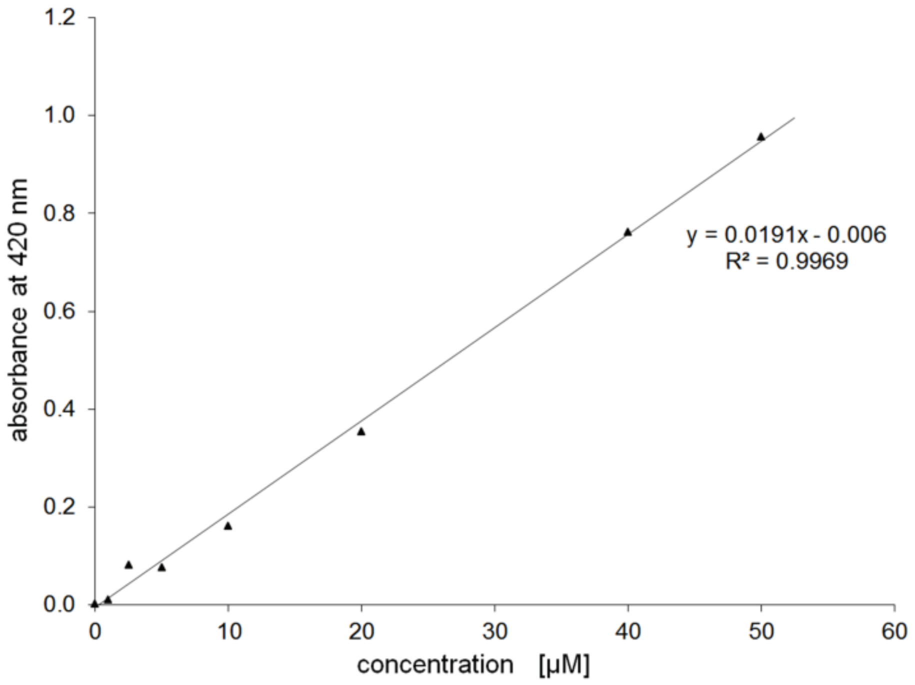

(**b**)

Figure S2. TNBSA-assay calibration curves with lysine hydrochloride (**a**) and bovine serum albumin (**b**). Chromogenic products (*N*-trinitrophenylamine) resulting from reaction of trinitrobenzene sulfonic acid (TNBSA) with primary amine (ε-amino group of lysine) were quantified in microplate reader at 420 nm. For calculating the modification degree of the neo-glycoproteins, calibration with bovine serum albumin (**b**) was used.

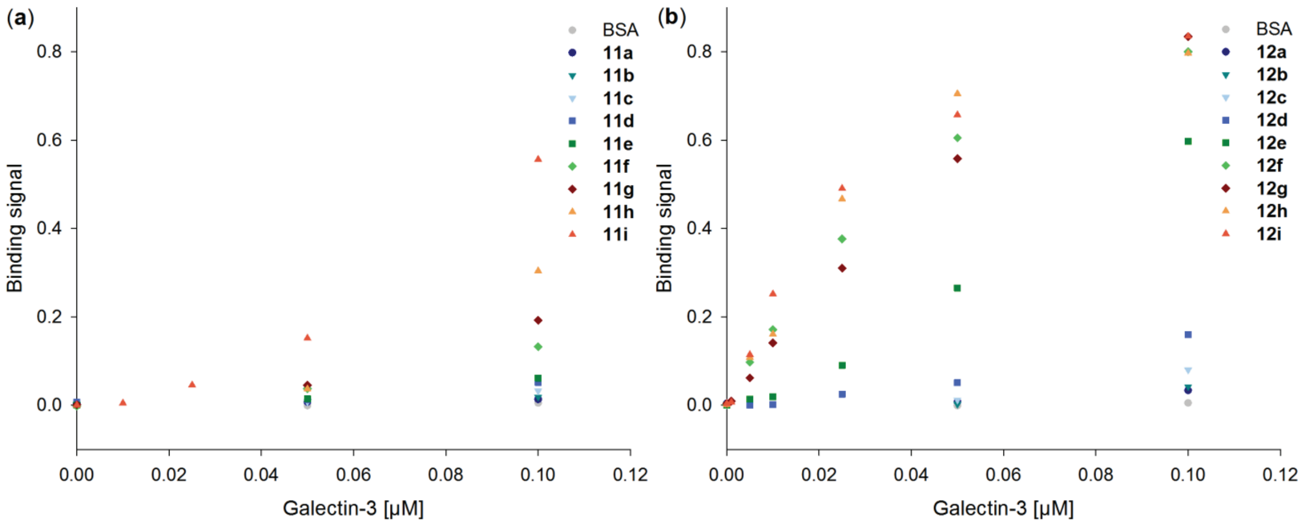

Figure S3. Binding signals of galectin-3 for concentrations of 0 to 0.1 µM to (**a**) LacNAc-LacNAc conjugated BSA (**11a**–**i**) and (**b**) LacDiNAc-LacNAc conjugated BSA (**12a**–**i**). Data were obtained for all neo-glycoproteins (**11a**–**i** and **12a**–**i**) in the galectin-3 concentration range of 0.05 to 10 µM. Moreover, binding signals of galectin-3 at concentrations below 0.05 µM were detectable for **11i** and **12d**–**i**.

Table S1. Binding signals of galectin-1 and galectin-3 to immobilized neo-glycoproteins **11a**–**i** and **12a**–**i**, ASF and BSA. Comparison of galectin-1 and galectin-3 binding is shown with standard deviation of at least 11 measured values. Significant higher binding of galectin-3 compared to galectin-1 is observed for neo-glycoproteins presenting LacNAc-LacNAc and LacDiNAc-LacNAc, respectively.

**Ligand**	**Binding Signal**		**Ligand**	**Binding Signal**	
	**Galectin-1**	**Galectin-3**		**Galectin-1**	**Galectin-3**
BSA	0.00 ± 0.01	0.00 ± 0.01	ASF	0.32 ± 0.05	0.28 ± 0.12
**11a**	0.02 ± 0.02	0.23 ± 0.04	**12a**	0.01 ± 0.01	0.21 ± 0.05
**11b**	0.02 ± 0.03	0.20 ± 0.04	**12b**	0.01 ± 0.01	0.39 ± 0.09
**11c**	0.06 ± 0.02	0.49 ± 0.04	**12c**	0.01 ± 0.02	0.54 ± 0.08
**11d**	0.13 ± 0.04	0.57 ± 0.05	**12d**	0.02 ± 0.02	0.68 ± 0.05
**11e**	0.23 ± 0.06	0.62 ± 0.05	**12e**	0.05 ± 0.03	0.74 ± 0.07
**11f**	0.31 ± 0.07	0.68 ± 0.04	**12f**	0.13 ± 0.05	0.85 ± 0.05
**11g**	0.34 ± 0.07	0.75 ± 0.06	**12g**	0.14 ± 0.06	0.95 ± 0.04
**11h**	0.34 ± 0.08	0.79 ± 0.05	**12h**	0.11 ± 0.06	0.90 ± 0.05
**11i**	0.40 ± 0.08	0.89 ± 0.05	**12i**	0.15 ± 0.06	0.95 ± 0.05

Table S2. K_d values of galectin-3 bound to neo-glycoproteins **11a**–**i** and **12a**–**i**. K_d in [µM] galectin-3 in ELISA-type binding assay to immobilized neo-glycoproteins (5 pmol) and respective standard deviations of at least 11 measured data are shown. Values were calculated by data fitting using equation for one site saturation ($y = \frac{B_{max} \cdot x}{K_d + x}$). Binding affinity of galectin-3 increases with increasing modification densities of neo-glycoproteins, more pronounced for LacDiNAc-LacNAc conjugated BSA (**12a**–**i**).

**Compound**	**K_d [µM]**	**Compound**	**K_d [µM]**
**11a**	0.86 ± 0.20	**12a**	0.33 ± 0.11
**11b**	0.97 ± 0.18	**12b**	0.46 ± 0.13
**11c**	0.69 ± 0.17	**12c**	0.45 ± 0.09
**11d**	0.63 ± 0.13	**12d**	0.23 ± 0.06
**11e**	0.52 ± 0.11	**12e**	0.08 ± 0.02
**11f**	0.30 ± 0.06	**12f**	0.03 ± 0.00
**11g**	0.21 ± 0.06	**12g**	0.04 ± 0.01
**11h**	0.18 ± 0.05	**12h**	0.03 ± 0.00
**11i**	0.11 ± 0.03	**12i**	0.03 ± 0.00

Table S3. Binding signals of galectin-3 per glycan of immobilized neo-glycoproteins **11a–i** (**a**) and **12a–i** (**b**) in relation to **11d** and **12d**, respectively. Binding signals at different galectin-3 concentrations are related to one binding site of the neo-glycoproteins and the relative potencies to **11d** or **12d** are given with standard deviations. For galectin-3 concentrations below 0.05 µM, binding to **12a–c** was not detectable (n.d.), setting binding signal per ligand for **12d** (and **11d**) to 1.00.

(a)

**Galectin-3 [µM]**	**Relative binding signal per glycan**								
	**11a**	**11b**	**11c**	**11d**	**11e**	**11f**	**11g**	**11h**	**11i**
10	2.76 ± 0.35	1.13 ± 0.13	1.37 ± 0.11	1.00 ± 0.08	0.73 ± 0.04	0.57 ± 0.03	0.47 ± 0.02	0.46 ± 0.02	0.43 ± 0.02
5	2.80 ± 0.39	1.15 ± 0.09	1.38 ± 0.10	1.00 ± 0.08	0.74 ± 0.04	0.56 ± 0.03	0.47 ± 0.02	0.46 ± 0.02	0.42 ± 0.02
1	2.53 ± 0.42	0.95 ± 0.18	1.41 ± 0.12	1.00 ± 0.10	0.76 ± 0.06	0.62 ± 0.04	0.55 ± 0.04	0.53 ± 0.03	0.54 ± 0.03
0.5	2.34 ± 0.39	0.88 ± 0.16	1.27 ± 0.19	1.00 ± 0.12	0.82 ± 0.08	0.77 ± 0.05	0.72 ± 0.05	0.73 ± 0.05	0.72 ± 0.04
0.25	1.60 ± 0.70	0.67 ± 0.25	1.10 ± 0.47	1.00 ± 0.31	0.96 ± 0.17	1.27 ± 0.09	1.42 ± 0.11	1.44 ± 0.11	1.56 ± 0.07
0.1	1.62 ± 0.96	0.94 ± 0.42	1.03 ± 0.62	1.00 ± 0.58	0.83 ± 0.36	1.32 ± 0.59	1.53 ± 0.98	2.26 ± 1.24	3.70 ± 0.64
0.05	3.58 ± 2.87	1.70 ± 1.78	0.94 ± 1.49	1.00 ± 0.97	0.76 ± 0.46	1.48 ± 0.72	1.43 ± 0.70	1.09 ± 0.81	3.98 ± 2.86

(b)

**Galectin-3 [µM]**	**Relative binding signal per glycan**								
	**12a**	**12b**	**12c**	**12d**	**12e**	**12f**	**12g**	**12h**	**12i**
10	1.35 ± 0.21	2.14 ± 0.28	1.18 ± 0.06	1.00 ± 0.06	0.68 ± 0.04	0.48 ± 0.02	0.45 ± 0.01	0.44 ± 0.02	0.40 ± 0.01
5	1.41 ± 0.26	2.18 ± 0.37	1.16 ± 0.10	1.00 ± 0.06	0.66 ± 0.04	0.47 ± 0.03	0.45 ± 0.02	0.42 ± 0.02	0.40 ± 0.01
1	1.62 ± 0.36	2.27 ± 0.52	1.18 ± 0.10	1.00 ± 0.07	0.68 ± 0.07	0.52 ± 0.03	0.51 ± 0.02	0.47 ± 0.03	0.45 ± 0.02
0.5	1.33 ± 0.40	1.62 ± 0.49	0.86 ± 0.10	1.00 ± 0.11	0.70 ± 0.09	0.55 ± 0.03	0.52 ± 0.02	0.48 ± 0.02	0.47 ± 0.01
0.25	0.75 ± 0.33	0.99 ± 0.36	0.59 ± 0.08	1.00 ± 0.15	0.86 ± 0.09	0.65 ± 0.04	0.63 ± 0.02	0.58 ± 0.04	0.56 ± 0.02
0.1	1.09 ± 0.62	1.05 ± 0.69	0.75 ± 0.23	1.00 ± 0.48	2.34 ± 0.25	2.07 ± 0.15	1.89 ± 0.15	1.76 ± 0.11	1.67 ± 0.09
0.05	0.77 ± 0.75	0.20 ± 0.93	0.35 ± 0.18	1.00 ± 0.58	3.26 ± 0.92	4.92 ± 0.85	3.96 ± 0.61	4.89 ± 0.55	4.14 ± 0.32
0.025	n.d.	n.d.	n.d.	1.00 ± 0.70	2.24 ± 0.90	6.16 ± 0.43	4.43 ± 1.48	6.50 ± 0.74	6.21 ± 2.31
0.01	n.d.	n.d.	n.d.	1.00 ± 5.58	10.01 ± 14.78	58.74 ± 15.82	42.18 ± 20.69	46.97 ± 15.05	66.87 ± 18.31
0.005	n.d.	n.d.	n.d.	1.00 ± 4.58	24.34 ± 13.11	115.27 ± 8.94	63.84 ± 7.09	109.12 ± 15.02	104.96 ± 11.61
